# Quantitative analysis of the cysteine redoxome by iodoacetyl tandem mass tags

**DOI:** 10.1007/s00216-017-0326-6

**Published:** 2017-04-07

**Authors:** Shakir Shakir, Joelle Vinh, Giovanni Chiappetta

**Affiliations:** ESPCI Paris, PSL Research University, Spectrométrie de Masse Biologique et Protéomique (SMPB), CNRS USR 3149, 10 rue Vauquelin, 75231 Paris cedex 05, France

**Keywords:** Redox proteomics, Cysteine oxidation, Tandem mass tags (TMT), Mass spectrometry

## Abstract

The redox conditions that reign inside a cell have a determining effect on a number of biological processes. Reactive oxygen species (ROS) and reactive nitrogen species (RNS) are key redox players and have been linked to a number of pathologies. They have also been shown to play an important regulating role in cell signaling events. On the proteome level, thiol groups of cysteinyl side chains constitute the major targets of ROS and RNS. A number of analytical techniques based on mass spectrometry have been developed to characterize the cysteine redoxome, often facing a number of technical challenges, mostly related to the lability, heterogeneity, and low abundance of the oxidized forms. Furthermore, any posttranslational modification (PTM) quantification method needs to take the parent protein’s expression level into account. While taking all these limitations into consideration, we have developed a quantitative analytical strategy named OxiTMT, based on chemical labeling with iodoacetyl isobaric tandem mass tags (iodoTMT). OxiTMT allowed the generation of quantitative redox data that could be normalized by the protein’s expression profile in up to three different conditions. The method was tested on *Escherichia coli* with or without an oxidative treatment. Results showed the method to be adequate for the analysis of cysteine PTMs with a good coverage of the cysteine redoxome, especially for the low abundant oxidized species. Some of the challenges that face reporter ion quantification of PTMs by mass spectrometry were also assessed. This study serves as a proof of concept of the established protocol and consequent data treatment step. The use of tandem mass tags opens the ways towards the application of the method to the study of tissues and sera.

**Electronic supplementary material:**

The online version of this article (doi:10.1007/s00216-017-0326-6) contains supplementary material, which is available to authorized users.

**Graphical abstract:**
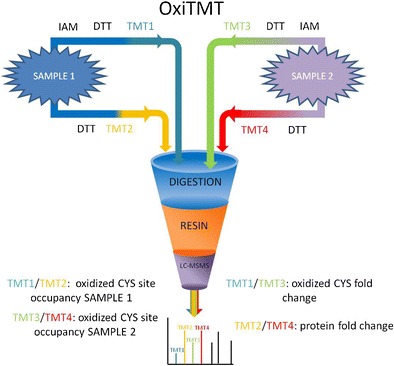
OxiTMT workflow

## Introduction

The accumulation of reactive oxygen species (ROS) in cells is regarded as a toxic event having a direct impact on a number of biomolecules. This event, referred to as oxidative stress, has been tied to aging and a variety of pathologies. Targets of ROS include DNA and lipids. However, proteins represent by far the largest fraction of affected family of molecules, with estimates placing that fraction as high as 70% [1]. At the proteome level, the presence of an increased amount of ROS leads to a number of posttranslational modifications (PTMs). Most notably, the oxidation of cysteine (Cys) residues leads to the formation of sulfenic acids (−S-OH), S-nitro groups (S-NO), and disulfides bridges, the latter known as oxidative folding [2]. With the discovery of cellular pathways using “cysteine switches” to mediate signal transduction in an analogous manner to phosphorylation, the way we view ROS has shifted from purely toxic compounds to important second messengers.

These general conclusions have led to the development of analytical techniques in proteomics aiming to exhaustively characterize and quantify PTMs on Cys residues. Redox proteomics remain a technical challenge due to the labile nature of thiol-redox reactions. Furthermore, when compared with other PTMs, the number of modified residues per protein can be high. The low abundance of oxidized proteins in the cytoplasm, combined to the intrinsic heterogeneity of the oxidized forms is another source of complexity. To date, the most efficient redox proteomics approach relies on differential labeling of cysteines according to their redox status and the subsequent identification of modified residues by a variety of techniques such as fluorescence or mass spectrometry. Efforts have also been made to go beyond identification in an aim to quantify the changes on a given residue. Among these strategies, OxICAT relies on heavy and light ICAT reagents as the two different alkylating agents to obtain a good estimation of the percentages of oxidized and reduced forms [3]. Unfortunately, this method does not allow obtaining simultaneous quantitative protein profile information. Moreover, the enrichment of the more abundant reduced fraction of the proteome could hinder the detection of oxidized cysteines.

Tandem mass tags (TMT) have been successfully used in numerous proteomics studies [4]. This amine-specific reagent is designed to obtain isobaric peptide labeling; thus, the quantification is achieved in tandem MS via reporter ion intensity comparison. The advantage of this approach compared with MS-based quantification is the higher sensitivity level and the reduction of MS spectra complexity allowing sample multiplexing. Recently, cysteine-specific iodoacetyl isobaric tandem mass tags (iodoTMT) have been reported. IodoTMT has been employed in the study of nitrosylation and other reversible cysteine modifications [5, 6].

Here, we propose a new redox proteomics workflow, OxiTMT, based on differential thiol trapping using iodoTMT reagents and liquid chromatography–tandem mass spectrometry (LC-MS/MS) analysis. This approach allows not only the quantification of cysteine redox changes but also protein expression profile monitoring in cells, tissue, and organ biopsies. In this paper, we applied OxiTMT to obtain a quantitative snapshot of the redox changes in *Escherichia coli* cells treated with 1 mM H_2_O_2_ by comparison with untreated cells.

## Materials and methods

LC-MS-grade acetonitrile (Fisher Chemical), iodoTMTsixplex™ reagents, Immobilized Anti-TMT™ Antibody Resin (ThermoFisher Scientific), Tris-buffered saline (TBS), dithiothreitol (dTT), iodoacetamide (IAM), urea, octyl β-d-glucopyranoside, protease inhibitor complete, LB medium (Sigma-Aldrich), bovine sequencing-grade trypsin (Roche), trifluoroacetic acid (TFA), trichloroacetic acid (TCA), formic acid (Fluka), and *E. coli* XL1-Blue strain (Agilent Technologies) were used.

### Cell growth and harvesting

*E. coli* was grown aerobically in LB medium at 37 °C until an OD_600_ of 0.7 was reached. The culture was then divided into two equal parts and with one part being incubated with 1 mM H_2_O_2_ for 30 min at 37 °C, and the other part serving as control. The cells were washed three times with phosphate-buffered saline to remove the growing medium and added reagents. Then each part was divided again into two equal parts.

### IAM labeling efficiency

In order to optimize the IAM alkylation step, three protein extracts of 100 μg each were resuspended in denaturing buffer (urea 6 M, Tris 100 mM pH 8.1, 1% octyl β-glucopyranoside, protease inhibitors) containing either: (1) no IAM, (2) 100 mM final concentration of IAM, (3) 200 mM final concentration of IAM (150 μL, 1 h, 37 °C in the dark). One microliter of 10 mM Dylight 550 maleimide sulfhydryl-reactive dye in denaturing buffer (30 min, room temperature) was added to each extract, and 20 μL of each extract were then finally separated by SDS-PAGE (12.5%). Gels were examined for fluorescence: excitation at 557 nm and emission at 572 nm (30 s accumulation; ImageQuant LAS 4000, GE Healthcare Life Sciences).

### OxiTMT procedure

Cells lysis was performed by adding 20% aqueous TCA and vortexing. Proteins were then pelleted by centrifugation (13,000 rpm, 4 °C, 1 h) and washed three times with ice-cold acetone to remove excess TCA. Each sample was then suspended in 150 μL of denaturing buffer supplemented with IAM (final concentration 200 mM) to block free thiols. The reaction was carried out at 37 °C in the dark for 1 h. Excess IAM was then removed by TCA precipitation, and the protein pellet was washed with acetone as described above. The proteins from each sample were suspended in 150 μL denaturing buffer supplemented with dTT (final concentration 20 mM) to reduce oxidized thiols (37 °C, 2 h). Excess dTT was removed by TCA precipitation. The protein pellets were then suspended in 100 μL denaturing buffer supplemented each with the entire content of one iodoTMT reagent vial as follows:

**Table:** 

Fraction	Label
Control oxidized thiol content	iodoTMT-126 (iodoTMT1)
H_2_O_2_-treated oxidized thiol content	iodoTMT-127 (iodoTMT2)
Control entire thiol content	iodoTMT-129 (iodoTMT3)
H_2_O_2_-treated entire thiol content	iodoTMT-130 (iodoTMT4)

The reaction was carried out at 37 °C in the dark following the manufacturer’s instructions. BCA protein assay confirmed an equivalent overall protein concentration in each fraction. Fifty microliters of each fraction were collected and pooled (final volume 200 μL) while the remaining from each fraction was used for label-free LC-MS analysis to further control a correct mixture of the different fractions. Excess iodoTMT was then removed from the pooled mixture by TCA precipitation (300 μL). The protein pellet was suspended in 50 mM ammonium bicarbonate and trypsin digested at 37 °C overnight.

### Labeled peptide enrichment step

Following the digestion step, peptides were lyophilized and then dissolved in 100 μL TBS at pH7.4 and loaded on 200 μL of the manufacturer’s slurry anti-TMT resin. The TMT-specific antibody enrichment step was carried out following the manufacturer’s instruction (4 °C, overnight). The unbound fraction was collected in the flow through (the resin was washed five times with 500 μL TBS), while the retained fraction was eluted using four equivalent column volumes of elution buffer provided in the iodoTMT kit. Both unbound and enriched fractions were lyophilized and suspended in 0.05% aq. TFA/ACN 98:2 (*v*/*v*).

### Liquid chromatography–mass spectrometry analysis

Peptides were desalted on a C_18_ cartridge (Acclaim PepMap100, 5 μm particles, 300 μm i.d. × 5 mm length, ThermoFisher Scientific) at 15 μL/min flow rate in buffer A, 0.05% aq. TFA/ACN 98:2 (*v*/*v*) for 5 min, before being eluted to a C_18_ column (Acclaim PepMap100, C_18_, 3 μm particles, 75 μm i.d. × 50 cm length, ThermoFisher Scientific) at a flow rate of 220 nL/min using a gradient of buffer B, ACN/0.1% aq. FA 90:10 (*v*/*v*): 2–40% B in 170 min, then 40–50% B in 10 min. The eluted peptides were analyzed by a nano-ESI quadrupole-Orbitrap mass spectrometer (Q Exactive, ThermoFisher Scientific) with the following parameters: one full MS acquisition (resolution 70,000 at *m*/*z* 200, AGC target value 1e6, max. injection time 250 ms), followed by ten MS/MS (TOP 10) with isolation window 2 *m*/*z*, fixed first mass 100 *m*/*z*, resolution 17,500 at *m*/*z* 200, AGC target value 5e4, max. injection time 120 ms, CE 30, and dynamic exclusion of 30 s.

### Data analysis

Protein identification was performed using MaxQuant (version 1.5.2.8) with the following parameters: reporter ion MS2 (iodoTMT 6plex), trypsin specific (with up to two missed cleavages), database *E. coli* (Uniprot August 2015, 4305 protein sequences), methionine oxidation, and cysteine carbamidomethylation were selected as variable modifications, first search peptide tolerance 20 ppm, main search peptide tolerance 6 ppm, and MS/MS match tolerance 20 ppm. Quantitative data mining was performed using Perseus 1.5.1.6. Significantly changed protein expression profiles were determined using the outlier identification tool, significance A, which calculated the probability (*p* value) of obtaining a log ratio of at least this magnitude under the null hypothesis that the distribution of log ratios has normal upper and lower tails. Only outliers with at least 1.5-fold change and a *p* value <0.05 were considered.

### SDS-PAGE analysis

Fluorescence was revealed using an ImageQuant LAS4000 instrument (GE Healthcare Life Sciences), with excitation at 557 nm and emission detected at 572 nm (30 s accumulation). Images were processed using ImageJ processing software.

## Results

### The concept of OxiTMT

Iodoacetyl tandem mass tags have been reported as a new sixplex isobaric reagent for irreversible Cys labeling, enabling to perform quantitative proteomics analyses [5]. IodoTMT reagents contain a sulfhydryl-reactive iodoacetyl group, a mass normalizing spacer arm and a mass reporter. Like with other isobaric labeling techniques, peptide quantification is based on the estimation of the relative area under the peak of the corresponding reporter ions obtained following MS/MS fragmentation.

**Fig. 1:**
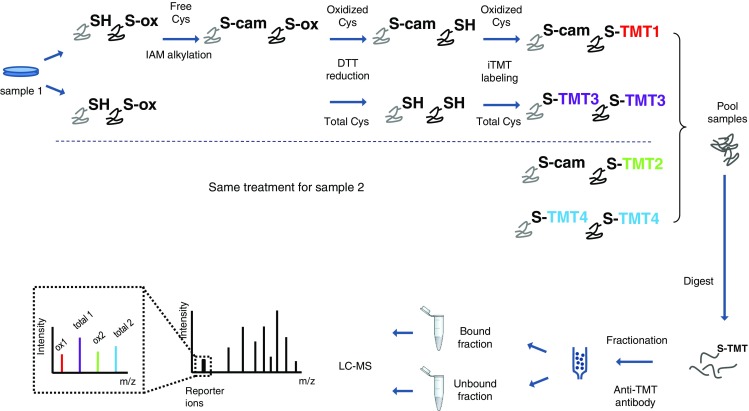
Schematic representation of the OxiTMT experimental workflow. This schematic illustrates the comparison of two samples. Both samples undergo the same labeling steps. In each case, a portion of the sample’s oxidized cysteine content is labeled with iodoTMT (TMT1 and TMT2 for samples 1 and 2, respectively) after blocking of free thiols with iodoacetamide (*IAM*), while in the other portion, the whole cysteine content is labeled with a different iodoTMT reagent (TMT3 and TMT4 for samples 1 and 2, respectively)

The idea behind the OxiTMT protocol (Fig. 1) was to divide each studied sample in two, where one part would have its total cysteine content labeled with an iodoTMT reagent whereas the second part would have solely its reversibly oxidized cysteine content labeled with a second iodoTMT reagent; in this case, reduced cysteines would have to be blocked first by an alkylating agent, iodoacetamide. The percentage of reversibly oxidized fraction for a given cysteine (%Ox = oxidized form abundance/total abundance) gives access to the information of the unmodified counterpart’s fraction. Since iodoTMT is available as a sixplex reagent, it is possible to compare up to three conditions in a single LC-MS run.

### Labeling efficiency

A crucial step in all redox proteomics studies is the initial step aiming to block the thiols of cysteine residues. This step is determinant to the generation of confident quantitative data. We tested a widely used alkylating agent: IAM.

To establish the optimal conditions, three protein extracts were subjected to different IAM blocking conditions: (1) no alkylating agent, (2) 100 mM final concentration of IAM, or (3) 200 mM final concentration of IAM. Reaction media were then supplemented with Dylight 550 maleimide sulfhydryl-reactive dye. Any residual free thiol from the IAM blocking step would react with the dye and be detected in the extracts by fluorescence following SDS-PAGE separation.

**Fig. 2:**
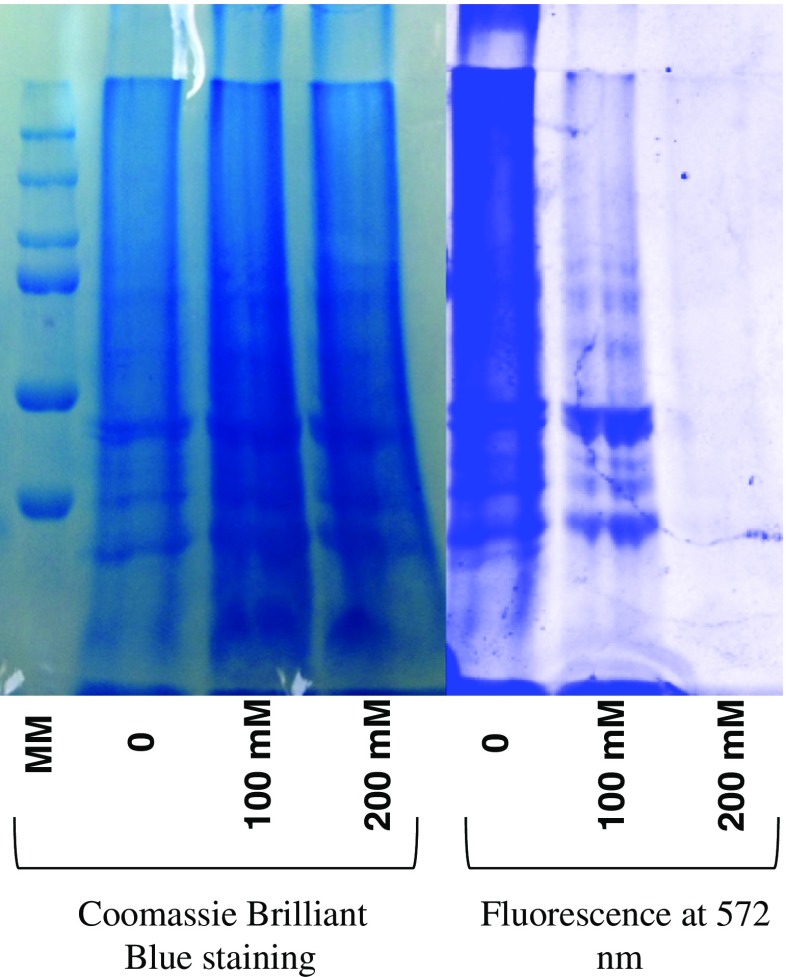
Labeling efficiency tested on three protein extracts. Fluorescence images were obtained by excitation at 557 nm, and the emission was detected at 572 nm (30 s accumulation). Brilliant Blue staining of the gel verifies the presence of proteins. The absence of fluorescence at 200 mM indicates an efficient alkylation of free thiols

Results show that at 200 mM of IAM (~5000 equivalents for each free thiol), no fluorescence is detected (Fig. 2). This concentration was adapted in our protocol for corresponding quantities of protein extracts.

**Fig. 3:**
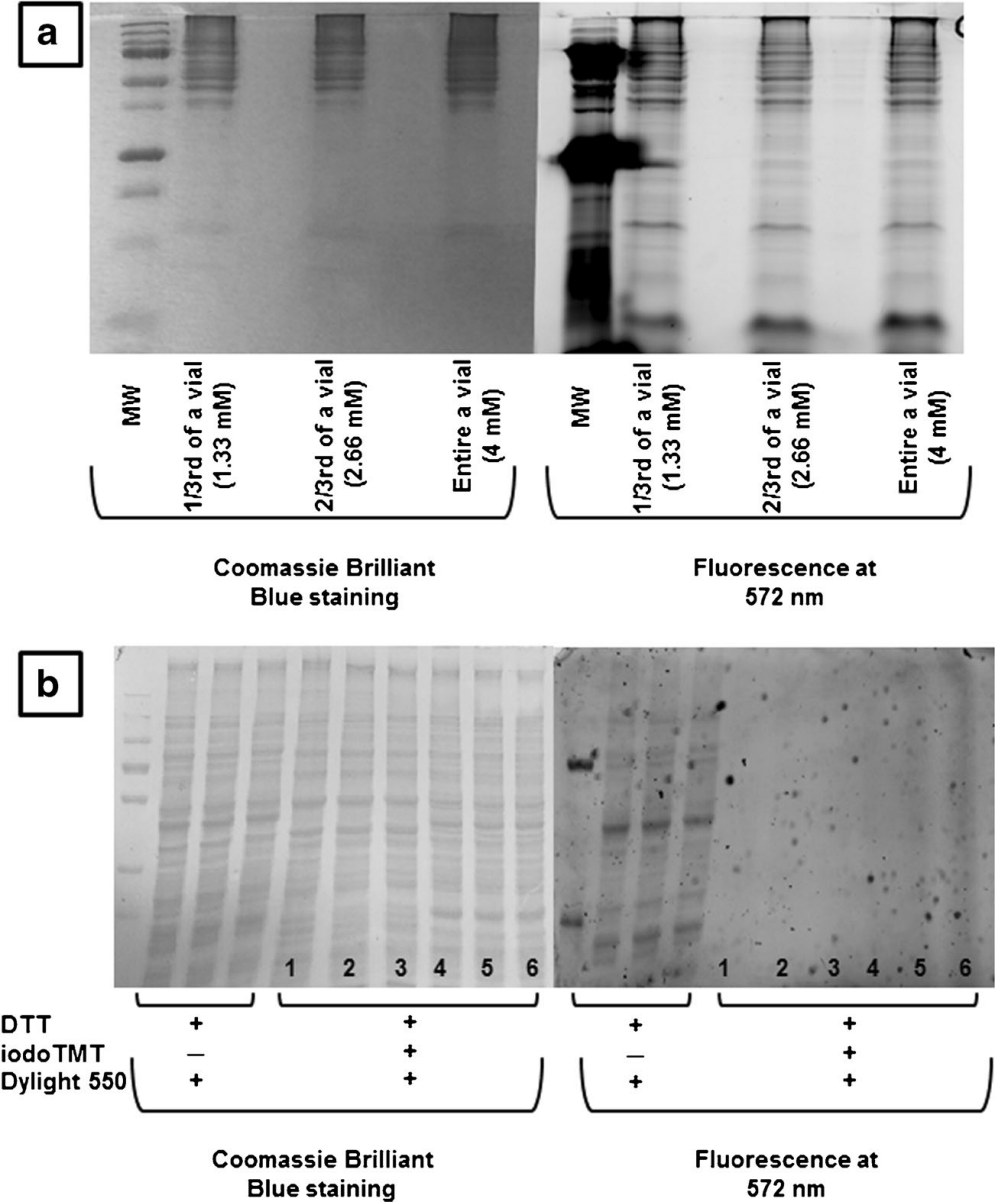
**a** IodoTMT tested as blocking agent for free cysteines. Labeling efficiency tested on three 100 μg protein extracts. The free cysteine residues in each extract were first blocked with either 1/3rd, 2/3rd or the entire content of an IodoTMT vial. At the end of the labeling reaction, Dylight 550 maleimide sulfhydryl-reactive dye was added to the media. Twenty-five micrograms of each protein extract were loaded and separated by SDS-PAGE. Gels were observed for fluorescence. Fluorescence images were obtained by excitation at 557 nm, and the emission was detected at 572 nm (30 s accumulation). Brilliant Blue staining of the gel verifies the presence of proteins. All lanes show residual fluorescence, showing that even the entire content of an IodoTMT reagent vial is not enough to block free cysteine residues. **b** IodoTMT labeling efficiency tested on 100 μg protein extract following reduction by dTT and denaturation. *Lanes 1 to 6* correspond to iodoTMT reagents 126 to 131, respectively. No fluorescence was detected following the alkylation step indicating the efficiency of alkylation with iodoTMT once proteins were reduced and denaturated. A control sample where the iodoTMT alkylation step was omitted shows that fluorescence was detected in this case

Previous studies have argued that one iodoTMT reagent can be used itself as a blocking agent for free cysteine residues while another isobaric version can be used to label reversibly oxidized cysteines upon their reduction [6]. This generates reporter ions for reduced and oxidized cysteines in an analogous way to the MS signal obtained for oxidized and reduced cysteines in OxICAT. However, a test on a 100-μg extract with the entire content of an iodoTMT vial (equivalent to a final concentration of 4 mM) showed that these conditions were not sufficient to extensively block free thiols (Fig. 3a). Using the theoretically necessary 200 mM of iodoTMT to block free thiols would be unrealistic with regard to the cost of a single experiment. This limitation does not apply to the blocking of initially oxidized cysteine thiols after reduction by dTT. Indeed, after reduction and denaturation, proteins lose their native conformation and are partially unfolded. Sterical hindrance decreases, and cysteine thiols are more accessible. On the opposite, blocking free cysteine requires a greater electrophilic moiety concentration because of the lower reactivity of reduced cysteine thiols in partially folded proteins. We tested the difference in cysteine thiol reactivity following reduction and denaturation on a 100-μg protein extract. Proteins were first reduced by dTT, TCA precipitated and then suspended in the denaturing buffer supplemented with one of the six available versions of the iodoTMT reagent (4 mM final concentration, iodoTMT to free thiol ratio ~70). Proteins were precipitated again and suspended in 50 mM ammonium bicarbonate supplemented with Dylight 550 maleimide sulfhydryl-reactive dye. No fluorescence was detected indicating the efficiency of alkylation by iodoTMT following protein reduction and denaturation (Fig. 3b).

### Quantification accuracy test

MS2 reporter ion quantification can suffer from compromised accuracy due to the unspecific co-isolation of neighboring *m*/*z* species during precursor ion selection. Moreover, compared to quantification strategies at the MS level (such as SILAC) that provides robust peak sampling, the robustness of quantification in MS2 increases with the number of times a given peptide is selected and sequenced. The lower the abundance of the species, the greater this effect, because of the dynamic exclusion that is used in classical proteomics data-dependent analyses.

In order to assess the accuracy of PTM reporter ion quantification by mass spectrometry, we chose three standard proteins with four cysteine residues each. The aim was to quantify the oxidized fraction in each of these proteins by submitting them to our protocol. In parallel, the oxidized fraction in these proteins is estimated by an independent method: non-reducing SDS-PAGE.

Measurements by mass spectrometry were done by submitting an equimolar mixture of bovine catalase (P00432), alkaline phosphatase from *E. coli* (P00634), and glyceraldehyde-3-phosphate dehydrogenase from rabbit (P46406) to our protocol as described above, labeling the oxidized fraction with iodoTMT-130 and the total cysteine content with iodoTMT-131. In order to take into account the complexity of the biological sample of interest, a complex proteins mixture from human cells was separately labeled with the same iodoTMT couple of reagents and was added to the three standard proteins and the mixture was digested and analyzed by LC-MS/MS. The oxidized ratio for each cysteine was obtained by calculating the ratio of 130/131 reporter ions.

Non-reducing SDS-PAGE experiments consisted in spotting 3 μg of each protein in two conditions: the untreated protein and the same amount of the protein after total reduction by dTT and alkylation by iodoacetamide. Gels were stained with Coomassie Brilliant Blue, and band intensities were estimated using ImageJ image processing software.

**Fig. 4:**
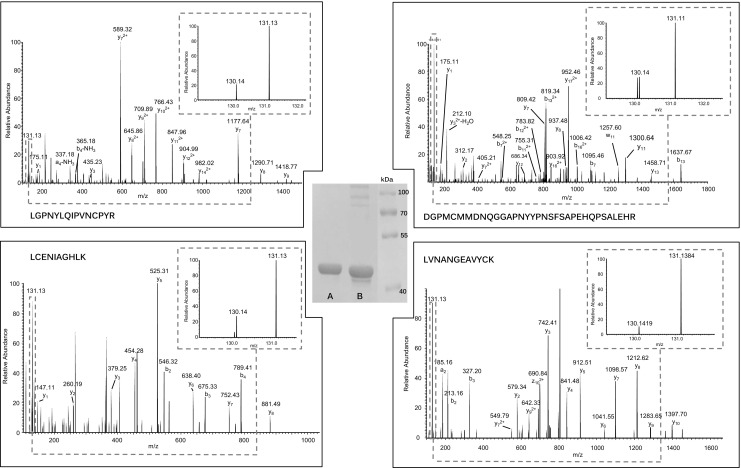
MS/MS spectra for each of the four cysteine containing peptides in catalase. *Center*, non-reducing SDS-PAGE bands for totally dTT-reduced catalase (*lane A*) and untreated catalase (*lane B*). The gel evidences more than one oxidized state of catalase in *lane B*: the monomeric oxidized form migrates underneath the reduced form, whereas multimeric oxidized forms migrate above. All these oxidized forms are reduced by dTT and comigrate in *lane A*. The MS/MS spectra for the four Cys-containing peptides reflect the relative quantification of oxidized state (130-reporter) vs. total peptide (131-reporter; see the *enlargement box at the upper right of each spectrum* for reporter ions relative intensities): LGPNYLQIPVNCPYR, 20%; LVNANGEAVYCK, 10%; LCENIAGHLK, 30%; and DGPMCMMDNQGGAPNYYPNSFSAPEHQPSALEHR, 30%. These spectra are consistent with the results obtained by non-reducing PAGE with the ImageJ software

It should be noted that, whereas OxiTMT provides accurate oxidized ratios for each cysteine individually, gel analyses reflect only the average oxidized ratio combination for all the cysteines in a given protein as widely reported in redox biology [7]. Providing distinct results for each cysteine using the OxiTMT protocol should reflect the overall trend observed by non-reducing SDS-PAGE. In Fig. 4, we report the results obtained for catalase. OxiTMT was able to provide the oxidized ratios for each of the four cysteines of the protein; these ratios ranged between 15 and 30%. Estimation of the oxidized fraction by non-reducing SDS-PAGE showed the protein to be 25% in the overall oxidized form in accordance with the findings by OxiTMT. The complete results for the remaining studied standard proteins are provided in the Electronic supplementary material (ESM) S1.

### *E. coli* redox proteomics analysis by OxiTMT

As a proof of concept for our quantitative redox strategy, we compared *E. coli* cells treated with 1 mM H_2_O_2_ for 30 min with *E. coli*-untreated cells. Total cysteine and oxidized cysteine fractions in each culture were labeled as explained above, and the proteome extracts were analyzed by LC-MS. In order to investigate the changes in the proteome and the redox state of the treated *E. coli* cultures, the data obtained from the MaxQuant quantification results were reprocessed by Perseus. Having two sets of information, i.e., the oxidized thiol fraction and the entire thiol content, the data can be processed to yield different sets of information. The ratio of the oxidized cysteine reporter ions between two conditions (iodoTMT2/iodoTMT1 in Fig. 1) reflects the change in the absolute quantity of the oxidized fraction of a given cysteine. This information needs to be corrected by the proteins expression level to distinguish changes due to redox phenomena from changes due to a simple variation of the protein’s expression level. To do so, the percentages of the oxidized fraction of a given cysteine can be obtained by calculating the quotient of the oxidized fraction’s reporter ion intensity to the total’s reporter ion intensity: iodoTMT1/iodoTMT3 for sample 1 and iodoTMT2/iodoTMT4 for sample 2 in Fig. 1. The ratio of oxidized cysteine percentages gives access to the change of the oxidized form of a given peptide reported to the total amount of this peptide in the sample of interest (sample 1 or 2) as opposed to the change in the amount of the oxidized peptide from samples 1 to 2 only. Finally, the ratio of the total cysteine content between two conditions (iodoTMT4/iodoTMT3, Fig. 1) gives access to the change in protein expression profile between samples 1 and 2, in the same way classic ICAT or iodoTMT quantitative experiments do. In practice, to obtain these different sets of data, we calculated the ratios in each analytical replicate. The values were then averaged, and the coefficients of variation (CV) were determined.

Since the two fractions of each sample received a different treatment, the samples were individually checked by label-free LC-MS analysis to ensure that equivalent overall protein contents (data not shown) were mixed. No reporter ion intensity correction was deemed necessary.

Search results allowed the identification of 1229 iodoTMT-labeled cysteines in total, associated to 580 proteins. Since the specificity of anti-TMT stationary phase is relatively low, we decided to take into account both bound and unbound fractions. In the bound fraction, 886 iodoTMT-labeled peptides (1019 cysteines) in 487 proteins were identified (of which 172 proteins were exclusively identified in this fraction). In the unbound fraction, 834 iodoTMT-labeled peptides (893 cysteines) in 408 proteins were identified (of which 93 proteins were exclusively identified in this fraction).

IodoTMT-labeled peptides represented 25% of the total identified peptides in the bound fraction (compared with 10% in the unbound fraction). If the depletion kit provided imperfect fractionation, it enhanced the detection of low abundance species, specifically in the bound fraction. All identified cysteines were either carbamidomethylated or iodoTMT labeled; and no unmodified cysteines were identified (with a posterior error probability <0.05 (calculated by MaxQuant)) in either fraction, validating thus the labeling efficiency.

Only cysteine containing peptides labeled with iodoTMT with reporter quantification in at least two technical replicates were considered for quantitative data mining. Profile expression ratios were determined as described above, and the values (log2 scale) were submitted to Perseus software. Significantly changed protein expression profiles were determined using the outlier identification tool, significance A [8], as described above. The test allowed the identification of 25 significantly changed expression levels (all up-regulated) in H_2_O_2_-treated cells (see ESM S2, Table S1). Up-regulated proteins belonged mostly to the oxidation reduction and generation of precursor metabolites and energy pathways (GOTERM_BP *p* value of 10^−5^ and 10^−4^, respectively, calculated with DAVID Bioinfomatic Tool [9]).

As for cysteine oxidation states, the percentages of the oxidized fraction in the treated cells were established as previously mentioned and then compared with the control by calculating the ratio: %Ox-treated cells/%Ox control cells (oxidized fraction in H_2_O_2_-treated cells (iodoTMT2/iodoTMT4)/oxidized fraction in control untreated cells (iodoTMT1/iodoTMT3). Outliers were determined using the same test performed on expression profiles with the maximum *p* value threshold being maintained at 0.05 and a minimum 1.5-fold change in the percentage of the oxidized fraction. We were thus able to detect a significant change in the redox state of 18 cysteine residues in the H_2_O_2_-treated cells. Unexpectedly, all of them showed a decrease of abundance in their oxidized fraction.

## Discussion

### H_2_O_2_-treated cell protein expression levels

Among the changes in protein expression levels in H_2_O_2_-treated cells, we found thioredoxin-1 (TrxA), thioredoxin-2 (TrxC), glutaredoxin-2 (GrxB), and glutaredoxin-3 (GrxC) to be up-regulated. All four proteins are involved in maintaining the redox homeostasis in *E. coli* cells. TrxA and TrxC belong to the thioredoxin-thioredoxin reductase pathway while GrxB and GrxC belong to the glutathione pathway. The up-regulation of these proteins is the cell’s response to the increase of H_2_O_2_ concentration due to the treatment. Peptide methionine sulfoxide reductase MsrB was also found to be up-regulated; MsrB reduces methionine sulfoxides to methionine and is regenerated by the Trx system (TrxA) [10]. Ferric uptake regulation (fur) protein is a global regulator involved in the oxidative stress response and is known to bind to TrxA [11]. RNA polymerase-binding transcription factor DksA also binds to TrxA, it is a 4-cysteine zinc-finger protein that has been described as a thiol switch for sensing oxidative and nitrosative stress [12].

Besides the proteins involved in the response to oxidative stress, proteins involved in anaerobic growth were found to be up-regulated: frdB and fdoH. It has been shown that oxidation of reduced thiols resulting from oxidative stress causes *E. coli* to switch to an anaerobic metabolism [13].

### H_2_O_2_-treated cells cysteine oxidation levels

In order to inspect the changes in the redox state of cysteine residues in treated cell, we calculated the ratio %Ox-treated cells/%Ox control cells and searched for outliers. This value reflects the combined effect of the change in the absolute quantity of an oxidized cysteine (Ox H_2_O_2_/Ox Ctrl) and the change in the parent protein’s expression level (expression profile). When examining these ratios (ESM S2, Table S2), different cases can be distinguished: (i) the significant change in the oxidized fraction results from a significant change in the quantity of the oxidized residue, while the protein’s expression profile remains unchanged, (ii) the significant change in the oxidized fraction is mainly due to a change in protein’s expression level, while the absolute quantity of the oxidized residue remains unchanged, and (iii) the significant change in the oxidized fraction is a combined effect of the two other values, which may be both significantly changed or not. OxiTMT, combining in a single analysis protein expression and PTM quantitative data, allows to distinguish different cellular events.

Our data show that most of the significantly changed redox states correspond to a decrease in the percentage of the oxidized fraction. This may seem paradoxical at first sight given the oxidizing nature of the treatment received by the *E. coli* cells. Upon inspecting the reduced cysteine residues, we focused the attention on TrxC peptide DDLPVVIDFWAPWCGPCR containing the protein’s redox active site (Cys64–Cys67) [14]. While the ratio of the absolute quantity of the oxidized residue has decreased (Ox H_2_O_2_/Ox Ctrl = 0.76), this change was not found to be significant. It is only when comparing the oxidized fraction relative with the protein expression profile (%Ox H_2_O_2_/%Ox Ctrl = 0.41) that this decrease in the H_2_O_2_ sample was found to be significant. Indeed, TrxC was found to be significantly up-regulated (1.8-fold change) in the H_2_O_2_-treated sample, indicating an increase in the expression of the protein mostly in its reduced form to counteract the effects of the oxidizing treatment. The burst of exogenous H_2_O_2_ leads to the activation of the anti-oxidant response that includes not only the thioredoxin pathway but also the glutathione pathway, leading to an adaptation to the stress situation and establishing a more reducing environment. Similar results, showing a general increase of the reduced fraction following an oxidative treatment, have already been reported and linked to an increase of the GSH/GSSG-reducing potential [15]. This was also observed with other studies using iodoTMT reagents to assess cysteine oxidation [6]. However, the authors did not assess protein expression profiles so the data cannot link the generally reduced state of the proteome to the up-regulation of thioredoxin pathway proteins.

**Fig. 5:**
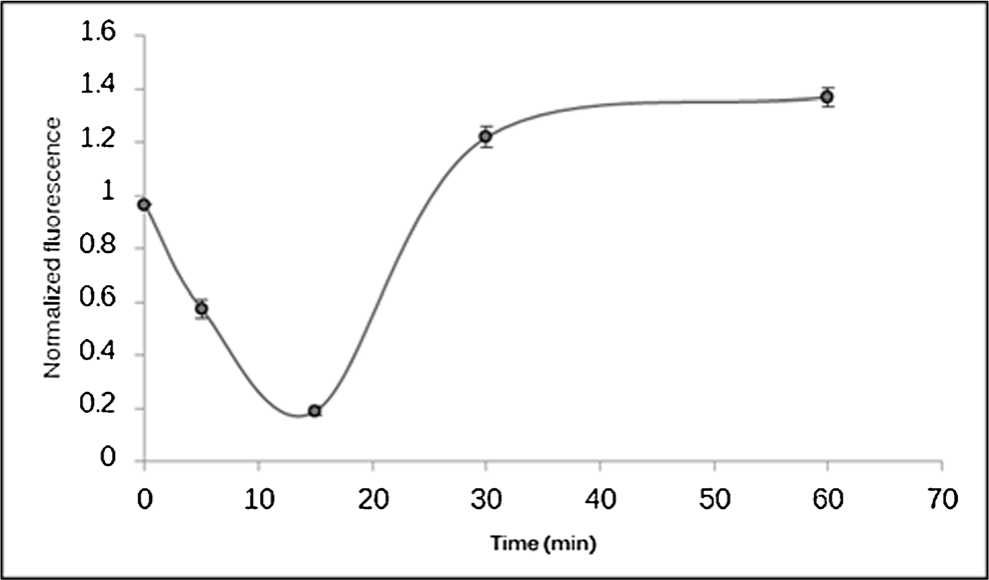
Kinetic plot representing the evolution of the total amount of free thiol following an exposure to 1 mM H_2_O_2_. The total fluorescence intensity measured for each lane at 572 nm was corrected by the total amount of protein in a given lane estimated by Coomassie Blue staining using ImageJ. The plot shows a rapid decrease of the amount of free thiol as expected due the presence of the oxidative reagent. The variation is quickly inversed after 15 min. The point at 30 min corresponding to the original OxiTMT experiment shows a higher amount of free thiol caused by the induction of anti-oxidant proteins as shown by proteomics results and appears as a plateau: this general reducing environment is maintained 60 min after the exposure to H_2_O_2_

We further tested the observation of a generally reduced environment following an oxidative treatment by submitting an *E. coli* proteome extract to a kinetic study. A 1-mM final concentration of H_2_O_2_ was added to 100 μg extracts. The reaction was stopped at different times by TCA precipitation. The quantity of free thiols was then estimated by Dylight 550 maleimide sulfhydryl-reactive dye labeling. Twenty micrograms of each time condition were separated by SDS-PAGE and revealed by fluorescence and Coomassie staining. The estimated free thiol quantity was corrected by the total protein amount in each lane in order to draw the kinetic plot (Fig. 5).

### Observations regarding the use of ion reporter MS for the quantification of PTMs

**Fig. 6:**
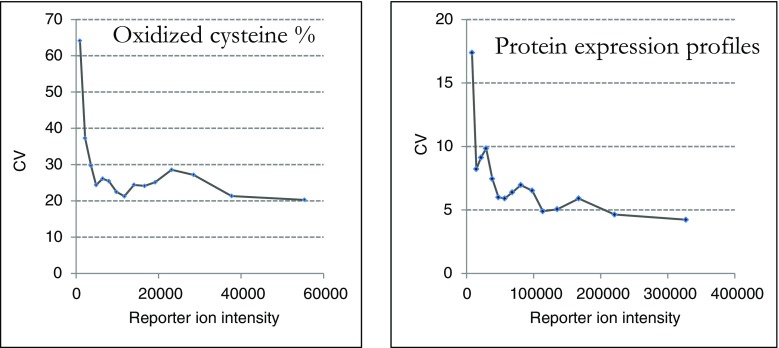
The two sets of generated data were affected differently as shown by the average CV as a function of reporter ion intensity. Oxidized cysteine percentages are calculated based on the oxidized cysteine reporter ion which is much prone to be affected by contamination by comparison with the more intense total cysteine reporter ion. The CVs for the oxidized fraction decrease with the reporter’s intensity, stabilizing under 30%, our limit of acceptance for a quantitative value

The present work aims at demonstrating the possibility of using iodoacetyl tandem mass tags in a multiplex redoxomic quantitative analysis strategy. The experimental design proved itself to be adequate to generate proper information: change in the oxidized fraction of a cysteine with direct correlation to the parent protein’s expression profile. The following step would be to develop and apply the strategy to the study of tissue and organ biopsies. The method, however, presents its own set of limitations. Indeed, as we were treating the data, we were faced with different challenges. One of them is inherent to reporter ion-based quantification by mass spectrometry. Co-isolation of peptides with close *m*/*z* values leads to the contribution of contaminant species leading to biased quantification of low abundance species. This implies that oxidized unabundant cysteine reporter ions are more affected than total cysteine reporter ions. This is reflected by the calculated coefficient of variation as shown in Fig. 6.

**Fig. 7:**
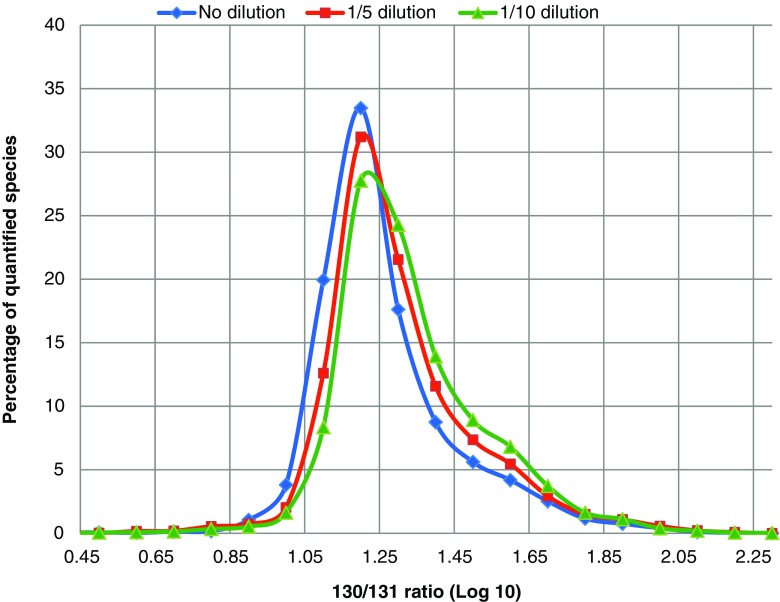
Distribution of 130/131 ratios for a 1:10 (*v*/*v*) mixture of two *E. coli* digests respectively labeled with TMT-130 and TMT-131 for three concentration ranges over 1 decade (no dilution, 1/5 dilution and 1/10 dilution)

We tested the effect of species abundancy on the accuracy of the quantification by submitting a mixture of wild-type *E. coli* iodoTMT-labeled proteomes to an LC-MS analysis. The idea behind this test is to consider a relatively extreme reporter ion ratio and to evaluate the threshold to accurately retrieve the correct ratio as the signal-to-noise ratio decreases. In short, two wild-type *E. coli* proteome extracts were labeled with iodoTMT130 and iodoTMT131 and then mixed 10 to 1. Three sample preparations were then analyzed by LC-MS: the 130/131 mixture alone (no dilution) and the 130/131 mixture spiked in an *E. coli* total unlabeled tryptic digest at either 1:5 (*v*/*v*) or 1:10 (*v*/*v*) (respectively 1/5 dilution or 1/10 dilution in Fig. 7). The distribution of the measured 130/131 ratio of the quantified species was then plotted in each case (Fig. 7). The theoretical distribution of quantified cysteine containing peptides should be centered around 1 (log 10 scale). While this is true for the undiluted mixture, a shift of experimental ratios towards higher values than the theoretical ones is observed as the dilution gets more important. A shoulder is observed in all three cases but is more important in the case of the 1/10 dilution.

Indeed, the noise is not negligible anymore for low abundance species. The accuracy measurement of low abundance ion reporters (TMT131) is more affected as its signal decreases. This underlines the specific challenge of ion reporter PTM quantification compared with protein quantification.

Another limitation of the method is that the calculation of the expression profiles is limited to cysteine-containing peptides. The higher the number of peptides used for the quantification, the more robust the quantitative information is. When performing protein quantification using several peptides, outliers (often peptides affected by multiple PTMs) are easily detected and eliminated. This advantage is unfortunately lost when the quantification is based on one peptide only. Very stringent criteria are required when quantifying PTMs (very low CVs, biological replicates are essential).

## Conclusion

In this paper, we reported a novel workflow, OxiTMT, for the simultaneous quantification of protein expression levels and oxidized cysteine residues. The iodoTMT reagent offers the possibility of comparing up to three different conditions in the same run. The method was tested on an *E. coli* model treated with hydrogen peroxide. Results showed the method to be adequate for the analysis of cysteine PTM with a good coverage of the cysteine proteome. The OxiTMT concept allowed the generation of redox data that could be normalized by the protein expression profile. This information is crucial and must be integrated to all PTM studies, regardless of the amino acid of interest. In our case, the absolute quantity of the oxidized cysteines in the active site of TrxC showed no significant variation, but the expression profile showed the protein to be significantly up-regulated, thus indicating a decrease of the overall oxidized fraction. This helped explain the reducing environment, generated by the cells to counteract the effect of the hydrogen peroxide treatment.

This study has also evidenced the different reactivity of cysteine thiols in folded and unfolded proteins, showing the necessity of optimizing reduced thiol saturation with less-expensive reagents such as IAM.

While this paper serves as a proof of concept for the developed method, OxiTMT’s potential benefits surpasses studies based simply on cell cultures. Indeed, contrary to metabolic labeling (SILAC), chemical labeling of proteins such as iodoTMT labeling offers the possibility to apply these methods to the study of tissue since protein labeling occurs after the extraction phase. This opens the door to varied fields of applications ranging from research to the development of diagnostic techniques.

## Electronic supplementary material

ESM 1 (PDF 1.10 mb)

ESM 2 (XLSX 45.6 kb)

ESM 3 (XLSX 125 kb)
