# Supplementary material for: Quantitative analysis of the cysteine redoxome by iodoacetyl tandem mass tags
Source: Anal Bioanal Chem. 2017 Apr 7;409(15):3821–30. doi: 10.1007/s00216-017-0326-6 (PMC5427158; doi:10.1007/s00216-017-0326-6)
Supplement: Supplementary file 1 — (PDF 1.10 mb) [file 216_2017_326_MOESM1_ESM.pdf]

Analytical and Bioanalytical Chemistry

Electronic Supplementary Material

Quantitative analysis of the cysteine redoxome by iodoacetyl tandem mass tags

Shakir Shakir, Joelle Vinh, Giovanni Chiappetta

Additional files available under “Supplementary Material”.

Content

ESM S1……………………………………………………………………………………..p. 3

ESM S1 contains the results of the quantification accuracy tests. For each of the three tested standard proteins fragmentation profiles of cysteine containing peptides are shown, as well as a zoom on the reporter ion region to evidence the ratio of reporter ion 130/131. This supplementary material also shows the non-reducing SDS-PAGE seperations performed on the three standard proteins as well as the quantification results obtained by ImageJ.

ESM S2………………………………………………………………………………………p. 17

ESM S2 contains two tables. Table S1 lists the significant changes in protein expression profiles in *E. Coli* cell cultures following the oxidizing treatment**.** Table S2 lists cysteine containing peptide sequences with significant changes in the oxidized fraction following the oxidizing treatment.

ESM S3 (see additional file)

ESM S3 provides a complete list of proteins quantified in the H_2O_2 treated cells vs. control cells.

ESM S4 (see additional file)

ESM S4 provides a complete list of cysteine residues quantified in the H_2O_2 treated cells vs. control cells.

ESM S1

Catalase OS=Bos taurus GN=CAT PE=1 SV=3 (P00432)

MS/MS Fragmentation of **DGPMCMMDNQGGAPNYYPNSFSAPEHQPSALEHR**
Found in **CATA_BOVIN**, Catalase OS=Bos taurus GN=CAT PE=1 SV=3

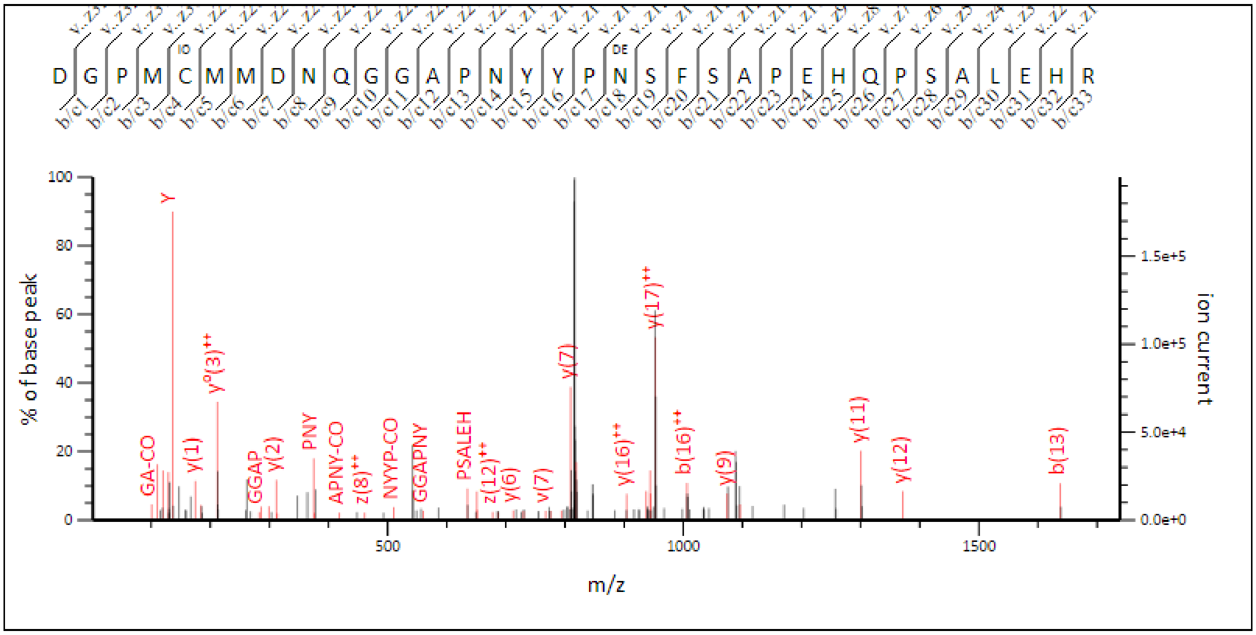

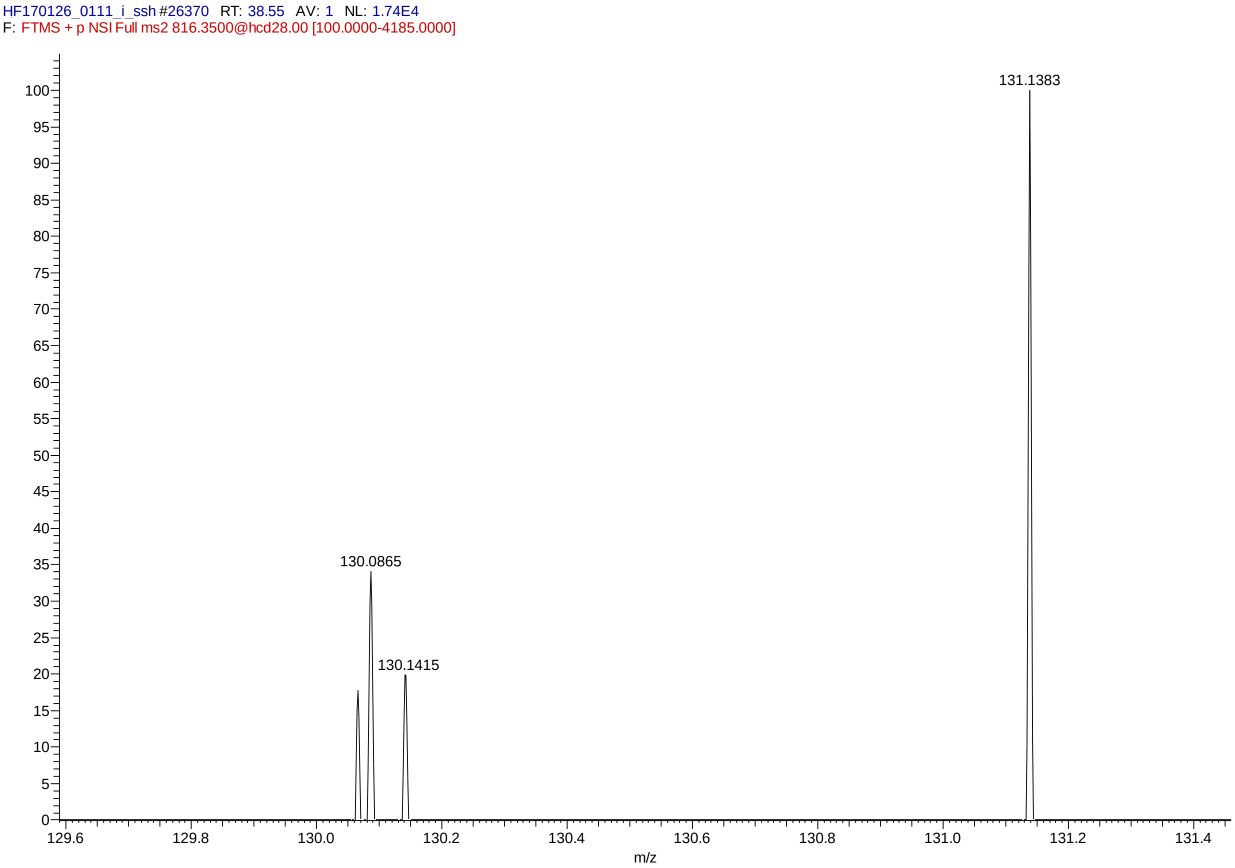

MS/MS Fragmentation of **LCENIAGHLK**
Found in **CATA_BOVIN**, Catalase OS=Bos taurus GN=CAT PE=1 SV=3

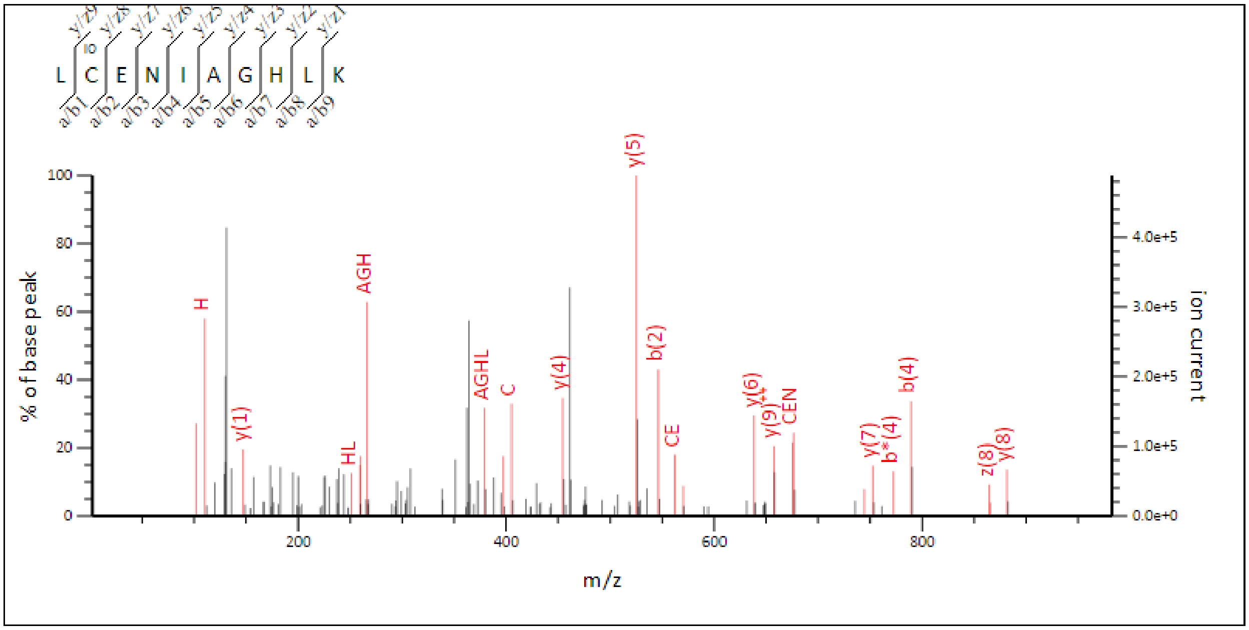

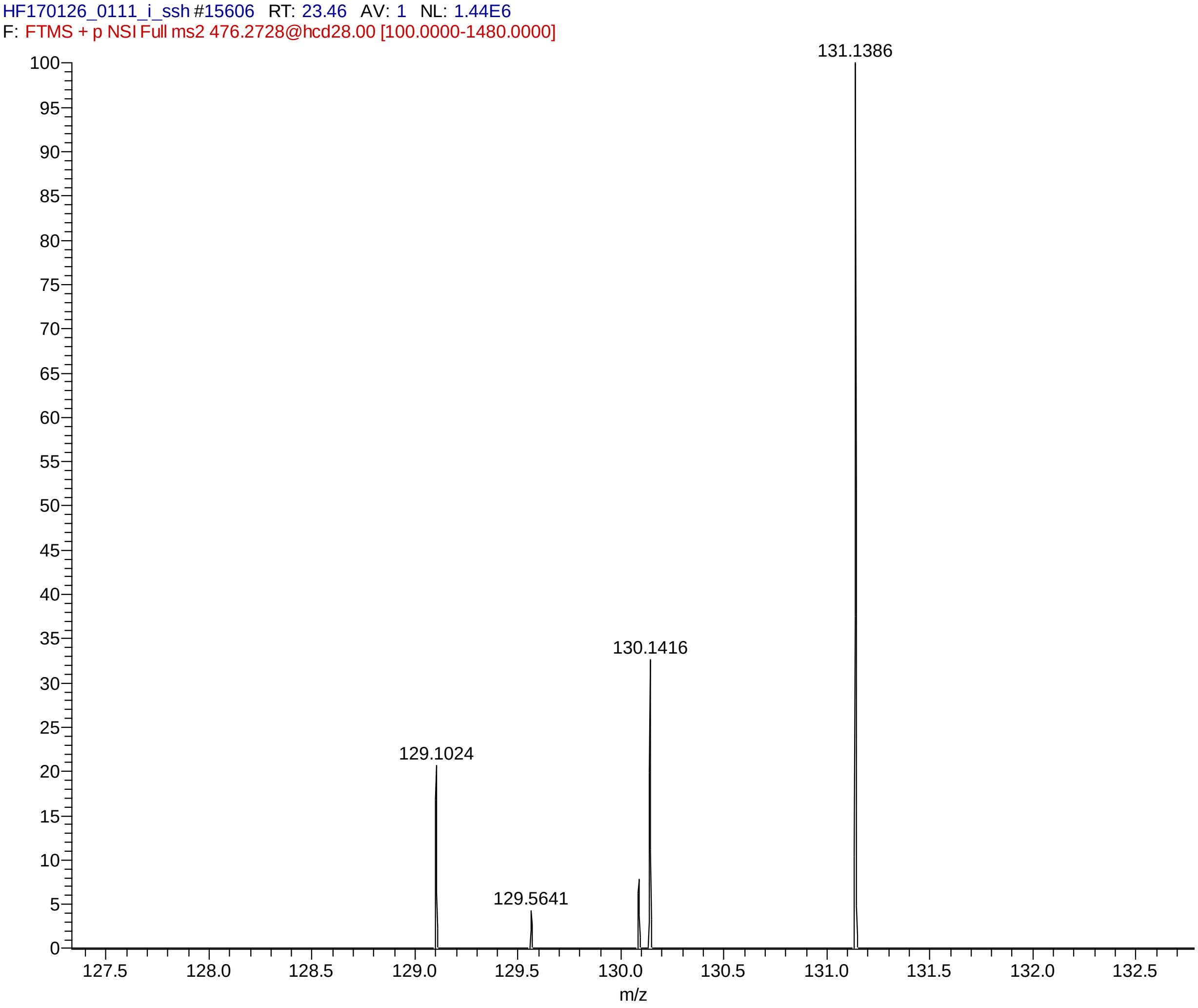

MS/MS Fragmentation of **LGPNYLQIPVNCPYR**
Found in **CATA_BOVIN**, Catalase OS=Bos taurus GN=CAT PE=1 SV=3

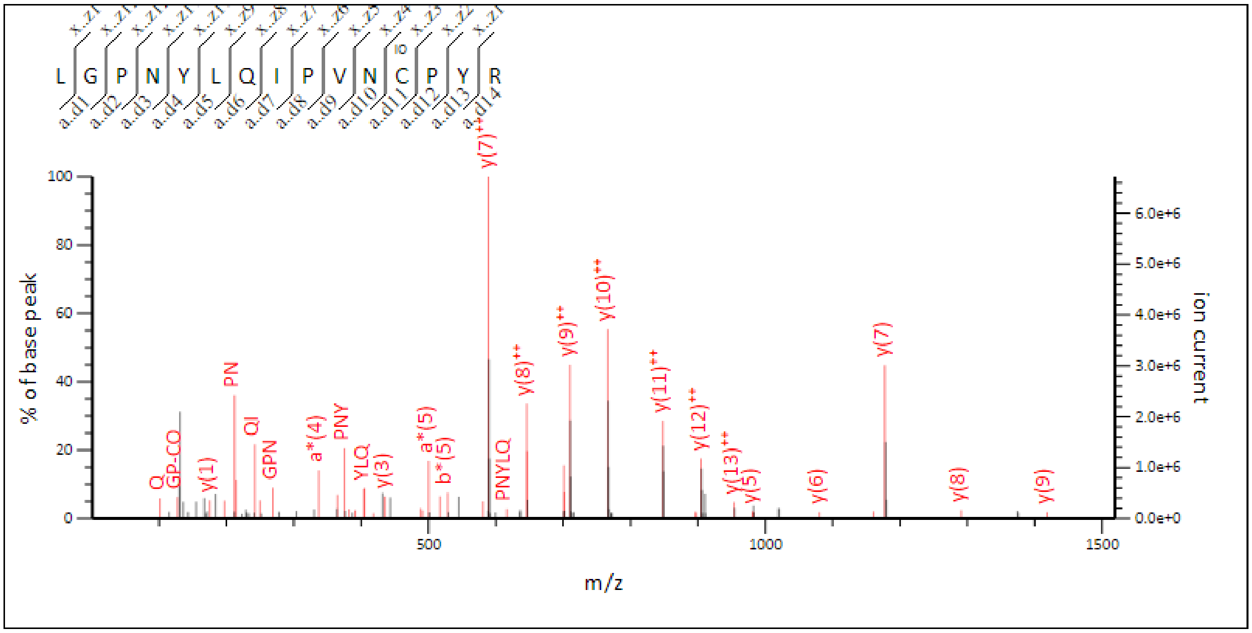

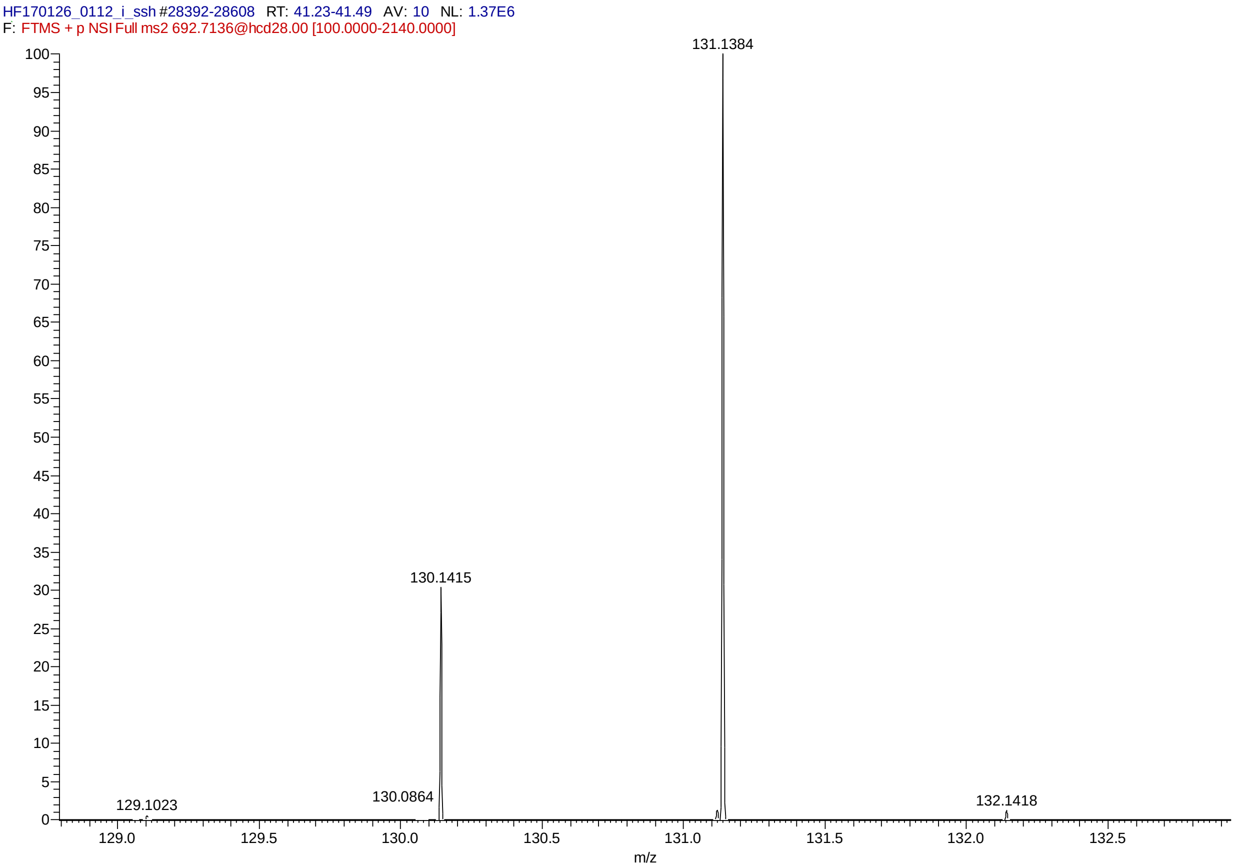

MS/MS Fragmentation of **LVNANGEAVYCK**

Found in **CATA_BOVIN**, Catalase OS=Bos taurus GN=CAT PE=1 SV=3

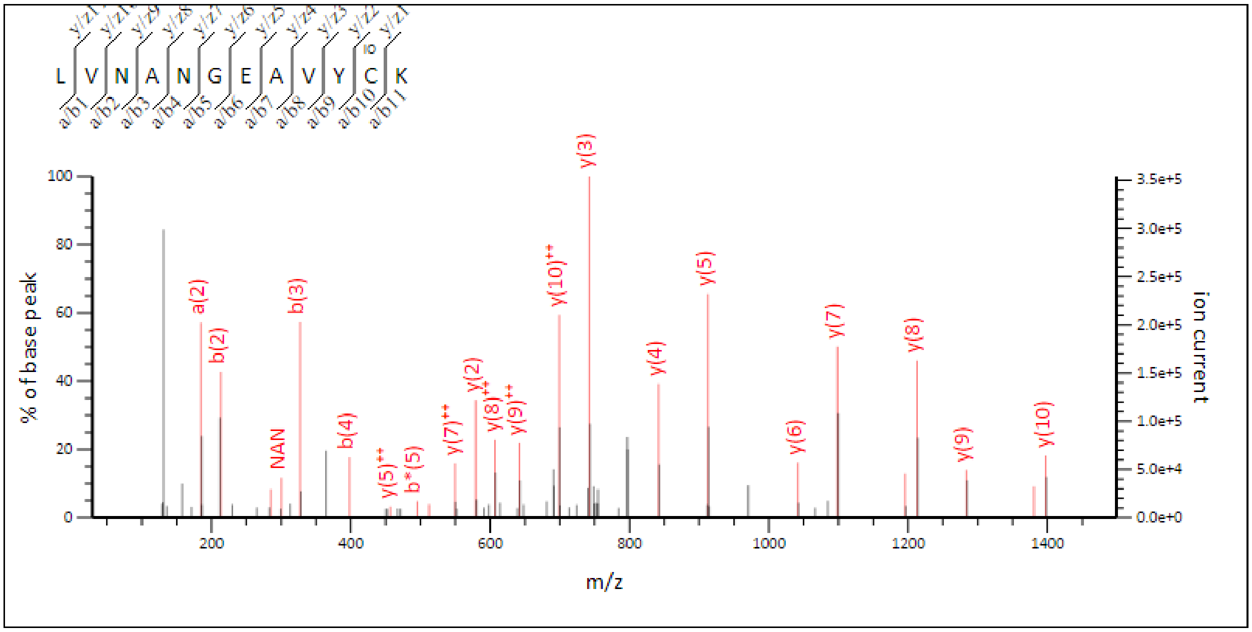

L V N A N G E A V Y C K
% of base peak
ion current
m/z
a(2)
b(2)
NAN
b(3)
b(4)
y(5)++
b*(5)
y(7)++
y(2)
y(8)++
y(9)++
y(10)++
y(3)
y(4)
y(5)
y(6)
y(7)
y(8)
y(9)
y(10)

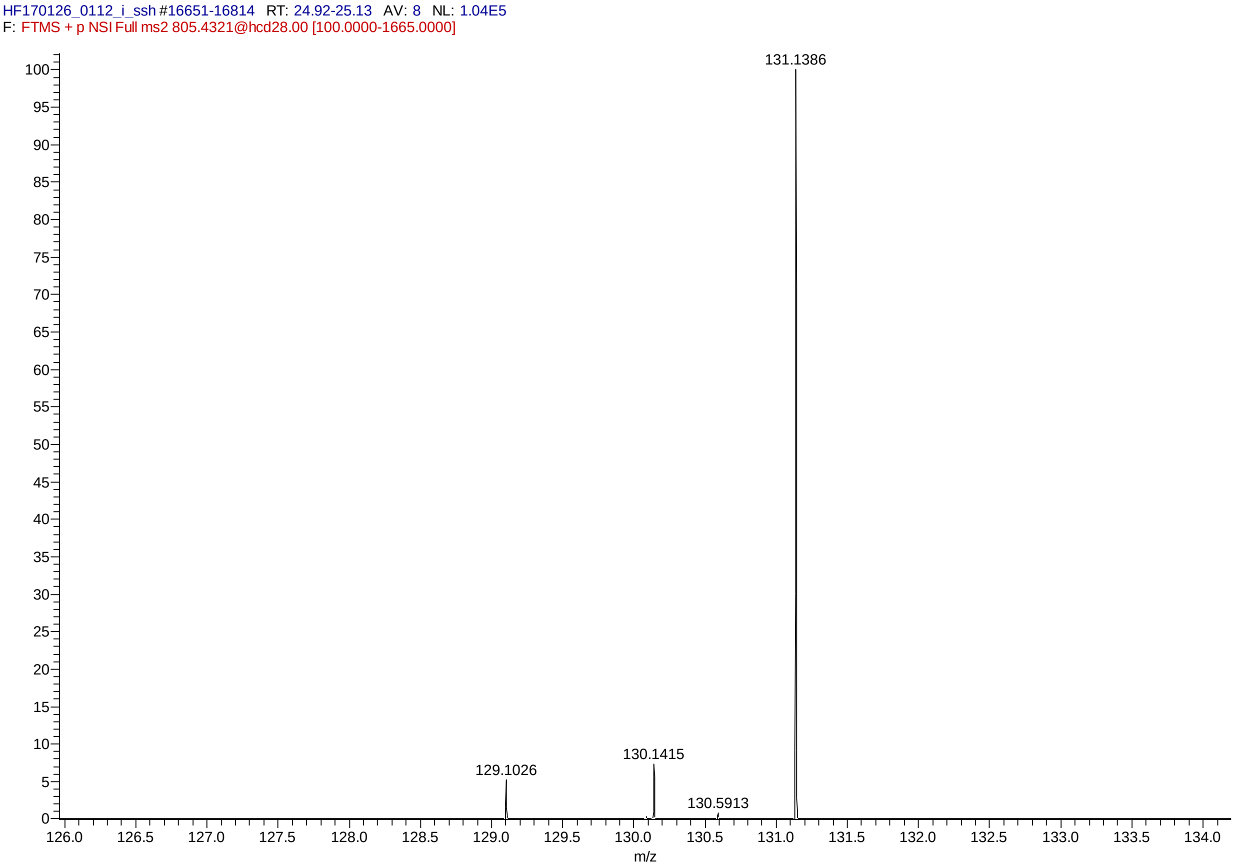

HF170126_0112_i_ssh #16651-16814 RT: 24.92-25.13 AV: 8 NL: 1.04E5
F: FTMS + p NSI Full ms2 805.4321@hcd28.00 [100.0000-1665.0000]
131.1386
129.1026
130.1415
130.5913
m/z

Alkaline phosphatase OS=Escherichia coli (strain K12) GN=phoA PE=1 SV=1 (P00634)

MS/MS Fragmentation of **ATYHGNIDKPAVTCTPNPQR**
Found in **PPB_ECOLI**, Alkaline phosphatase OS=Escherichia coli (strain K12) GN=phoA PE=1 SV=1

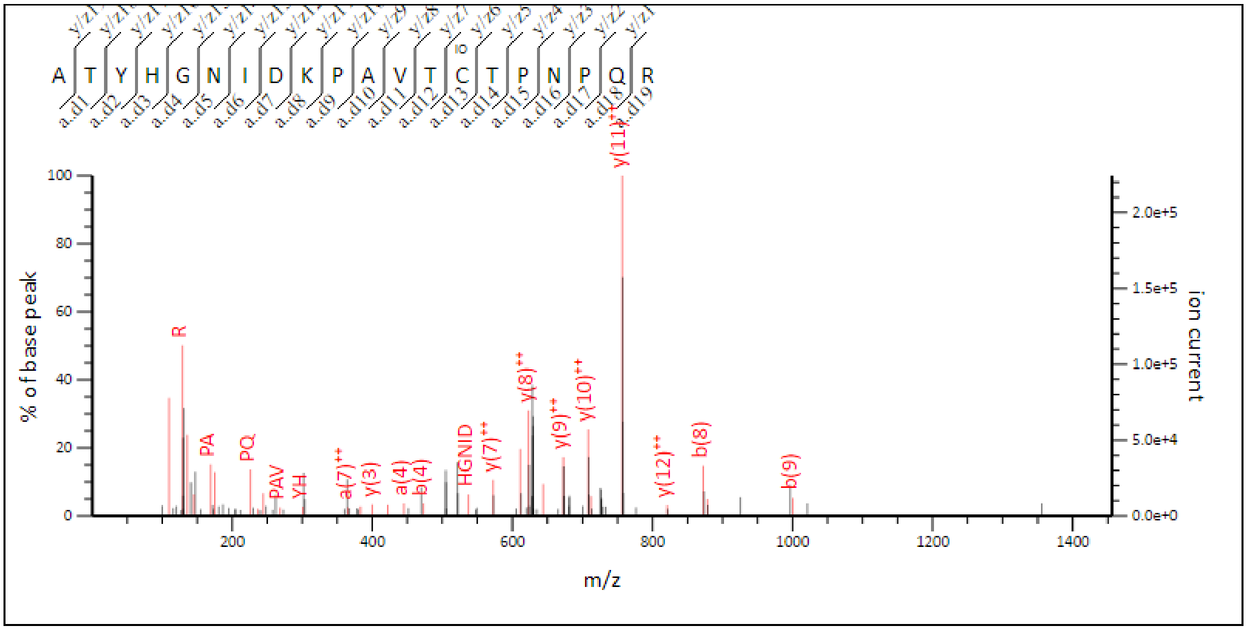

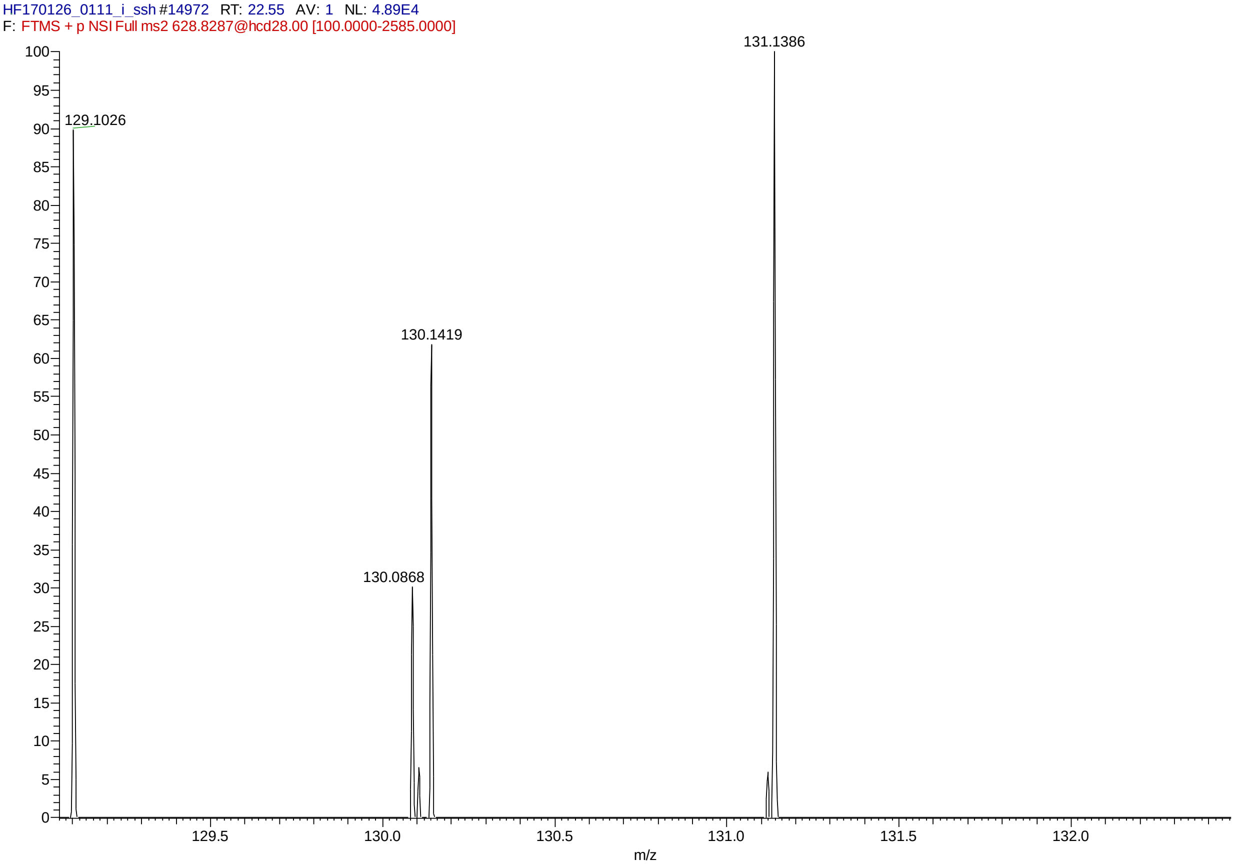

MS/MS Fragmentation of **CYGPSATSEK**
Found in **PPB_ECOLI**, Alkaline phosphatase OS=Escherichia coli (strain K12) GN=phoA PE=1 SV=1

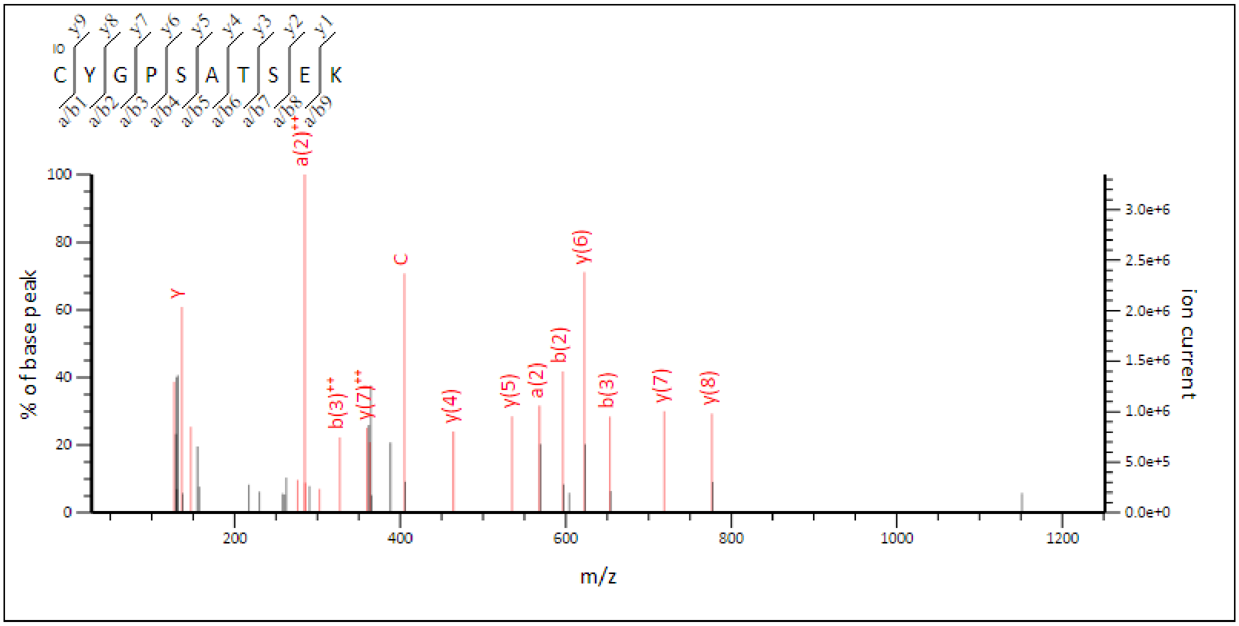

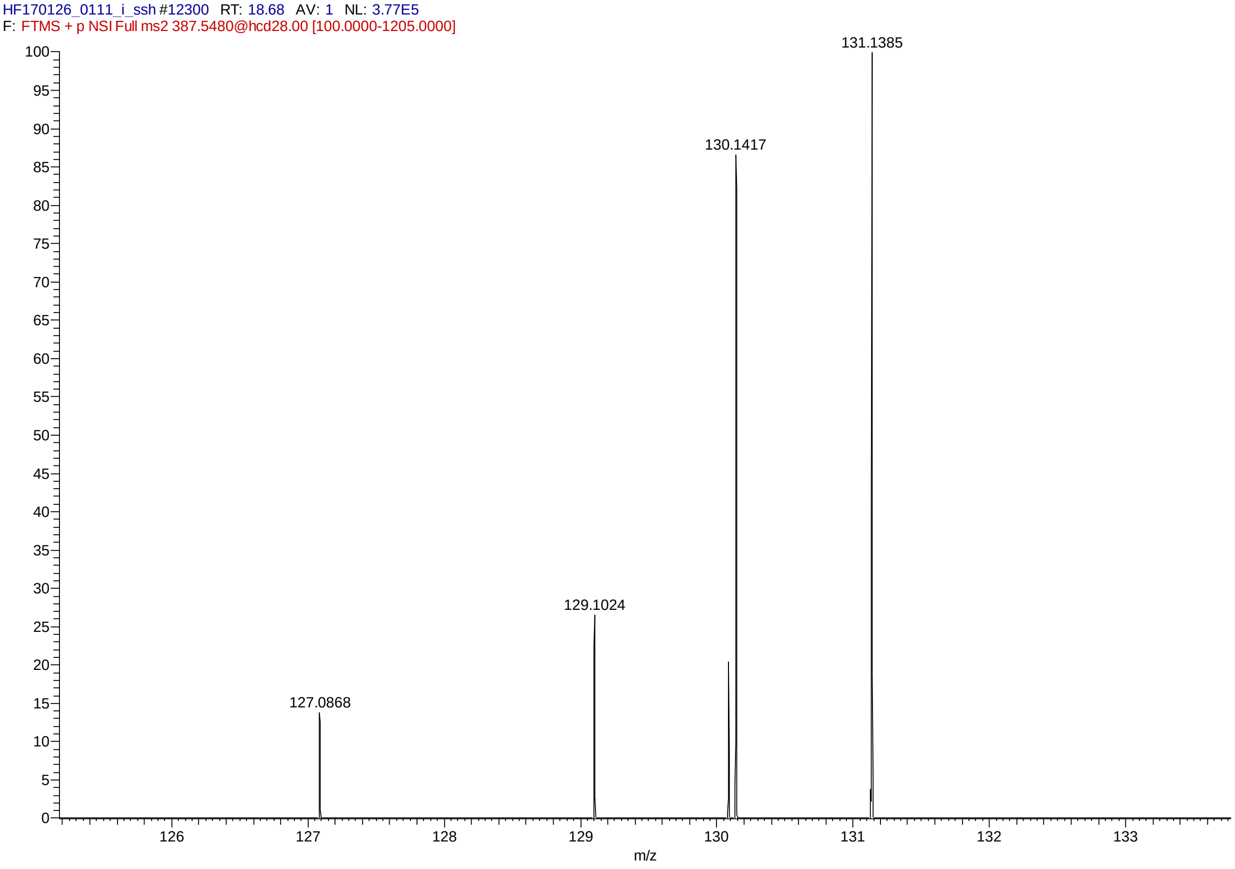

MS/MS Fragmentation of **CYGPSATSEK**
Found in **PPB_ECOLI**, Alkaline phosphatase OS=Escherichia coli (strain K12) GN=phoA PE=1 SV=1

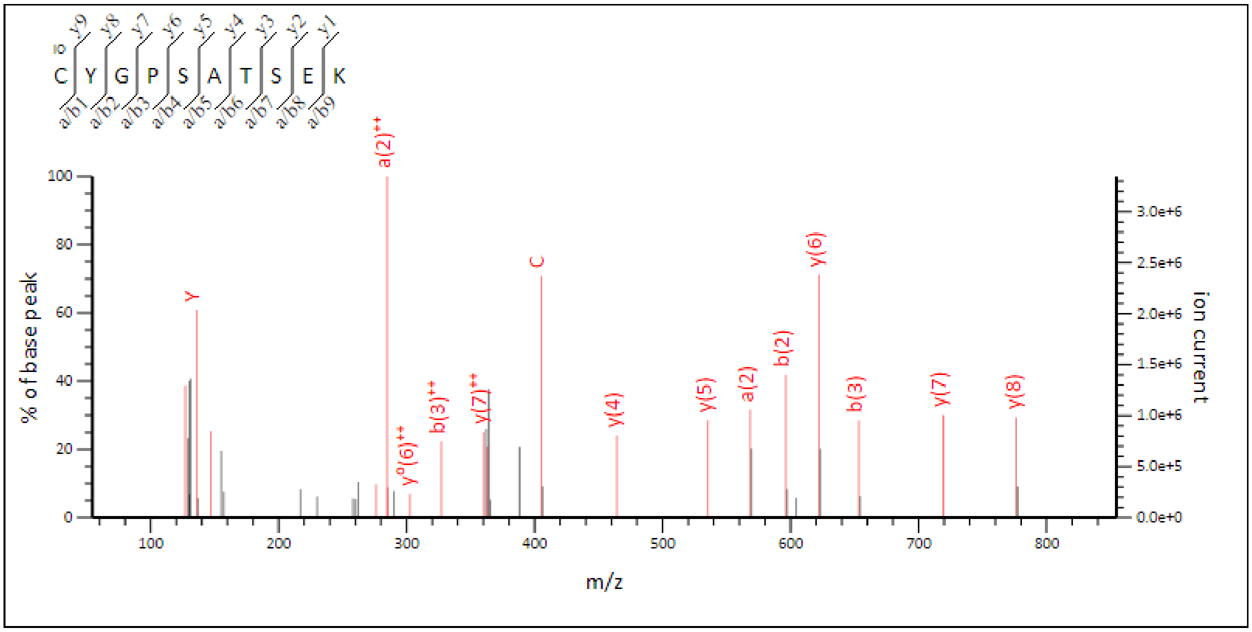

HF170126_0111_i_ssh #12528 RT: 19.00 AV: 1 NL: 3.59E5
F: FTMS + p NSI Full ms2 457.8974@hcd28.00 [100.0000-1420.0000]

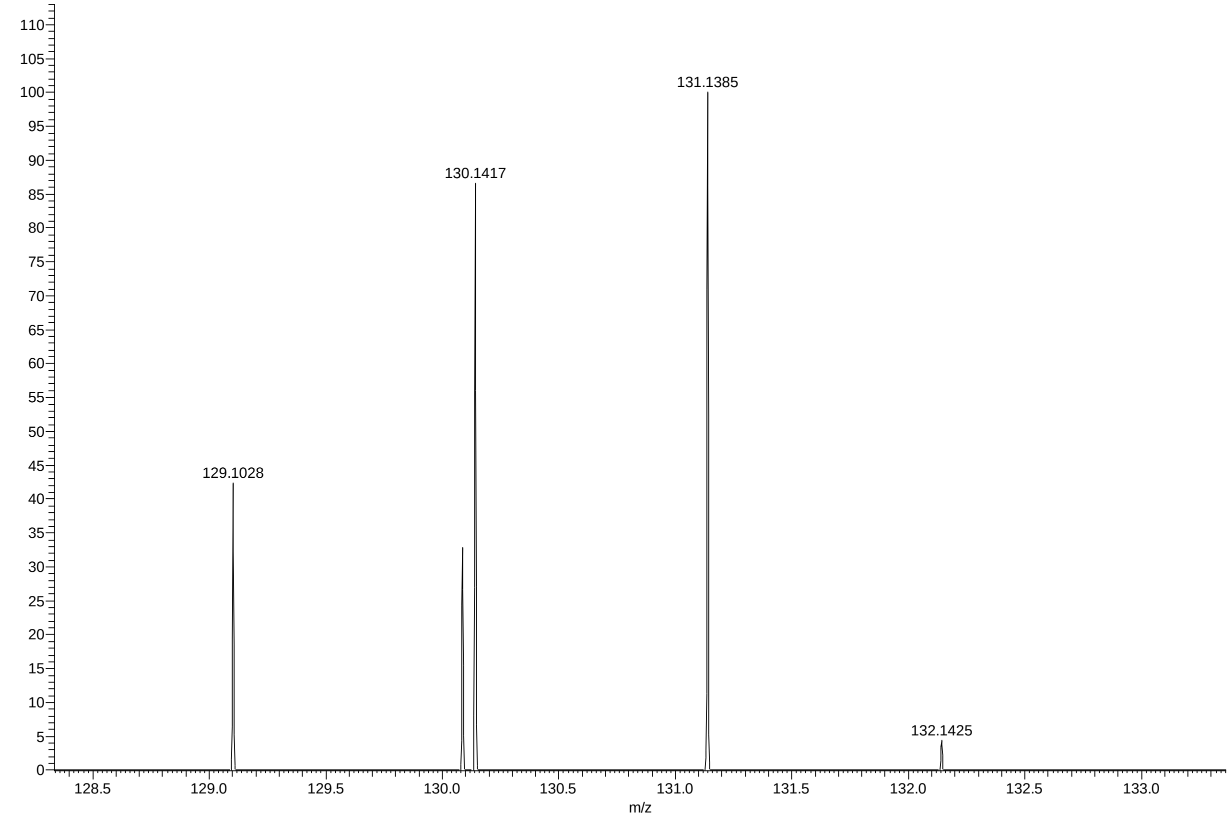

MS/MS Fragmentation of **QDHAANPCGQIGETVDLDEAVQR**
Found in **PPB_ECOLI**, Alkaline phosphatase OS=Escherichia coli (strain K12) GN=phoA PE=1 SV=1

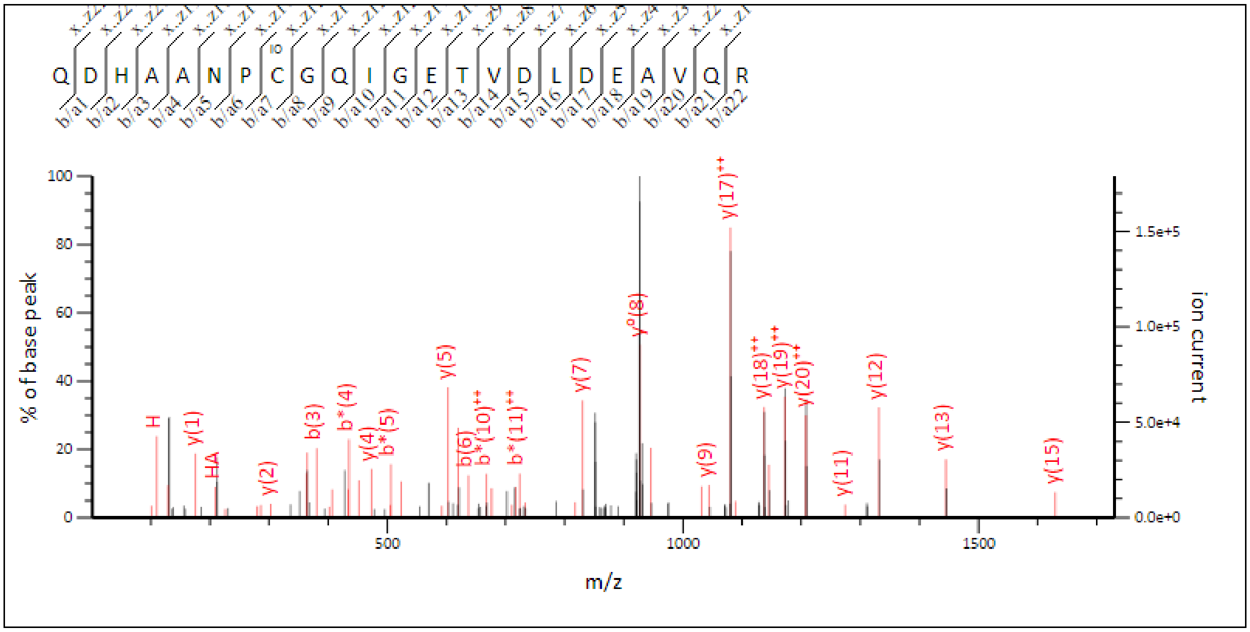
Q D H A A N P C G Q I G E T V D L D E A V Q R
% of base peak
ion current
m/z
H
y(1)
HA
y(2)
b(3)
b*(4)
y(4)
b*(5)
y(5)
b(6)
b*(10)++
b*(11)++
y(7)
y°(8)
y(9)
y(17)++
y(18)++
y(19)++
y(20)++
y(11)
y(12)
y(13)
y(15)

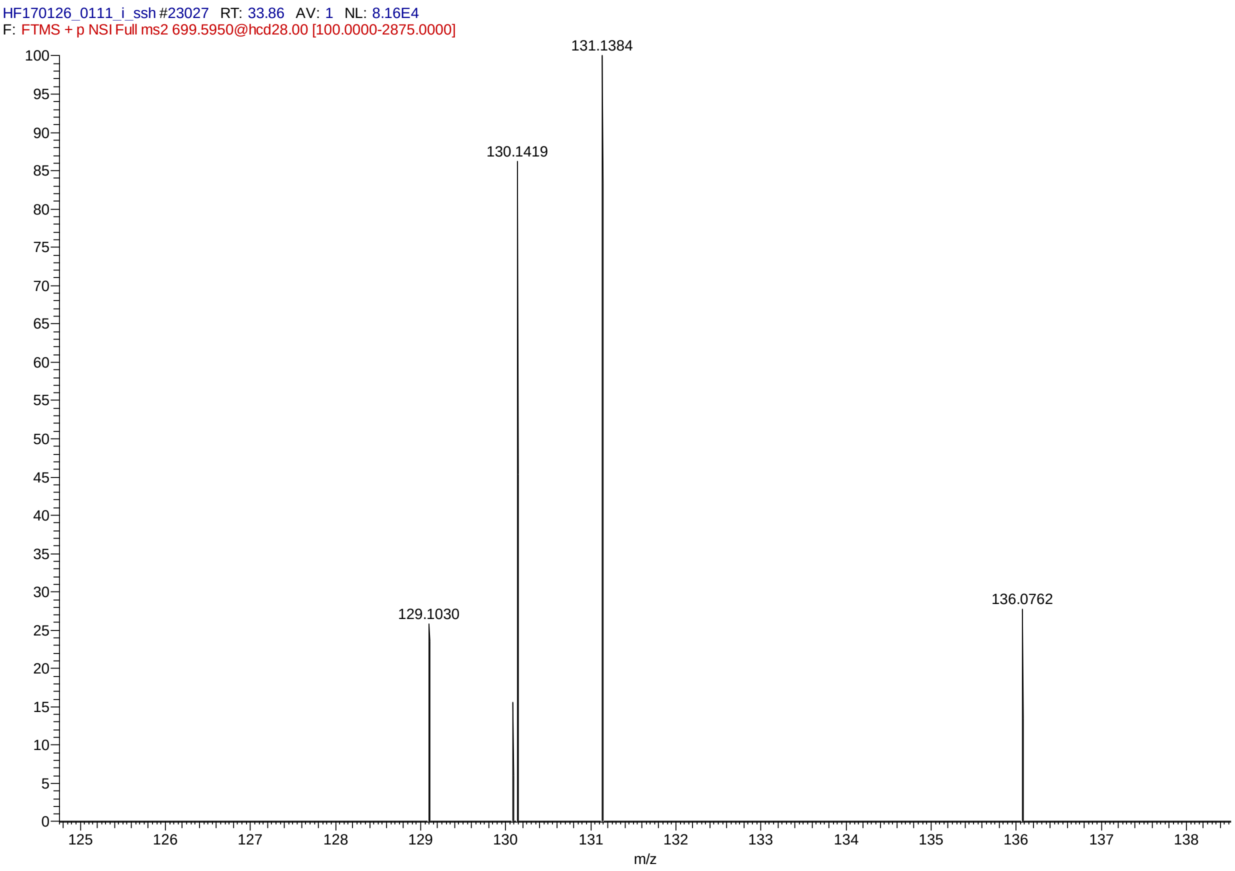
HF170126_0111_i_ssh #23027 RT: 33.86 AV: 1 NL: 8.16E4
F: FTMS + p NSI Full ms2 699.5950@hcd28.00 [100.0000-2875.0000]
131.1384
130.1419
129.1030
136.0762
m/z

Glyceraldehyde-3-phosphate dehydrogenase OS=Oryctolagus cuniculus GN=GAPDH PE=1 SV=3 (P46406)

MS/MS Fragmentation of **VPTPNVSVVDLTCR**
Found in **G3P_RABIT**, Glyceraldehyde-3-phosphate dehydrogenase OS=Oryctolagus cuniculus GN=GAPDH PE=1 SV=3

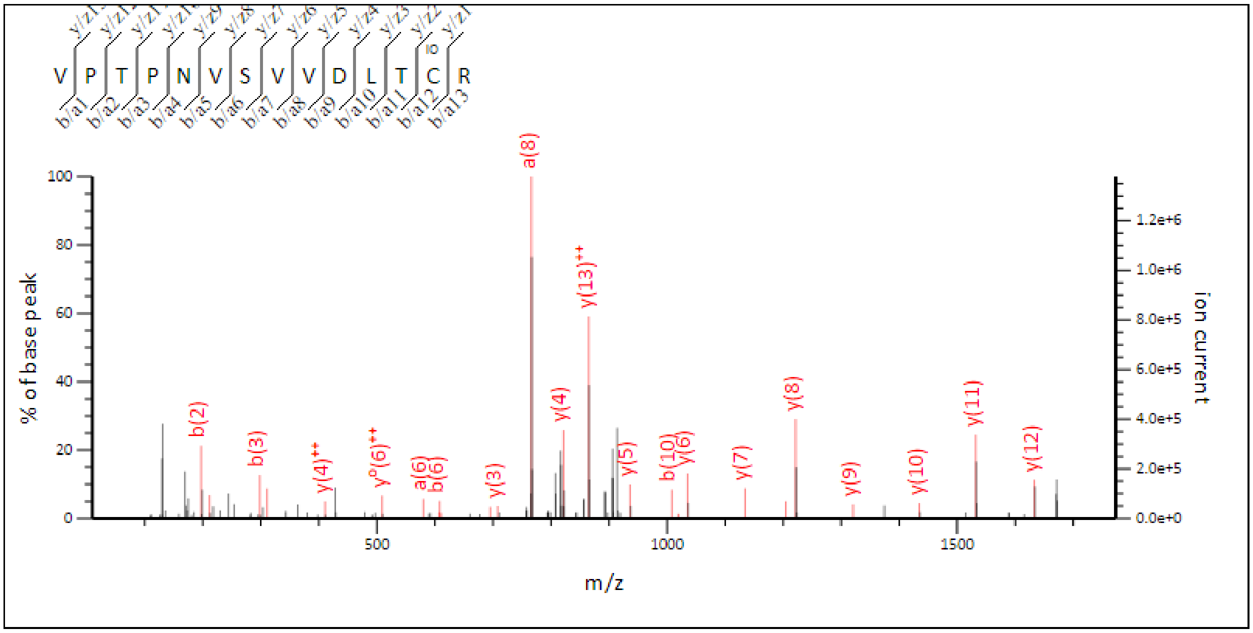

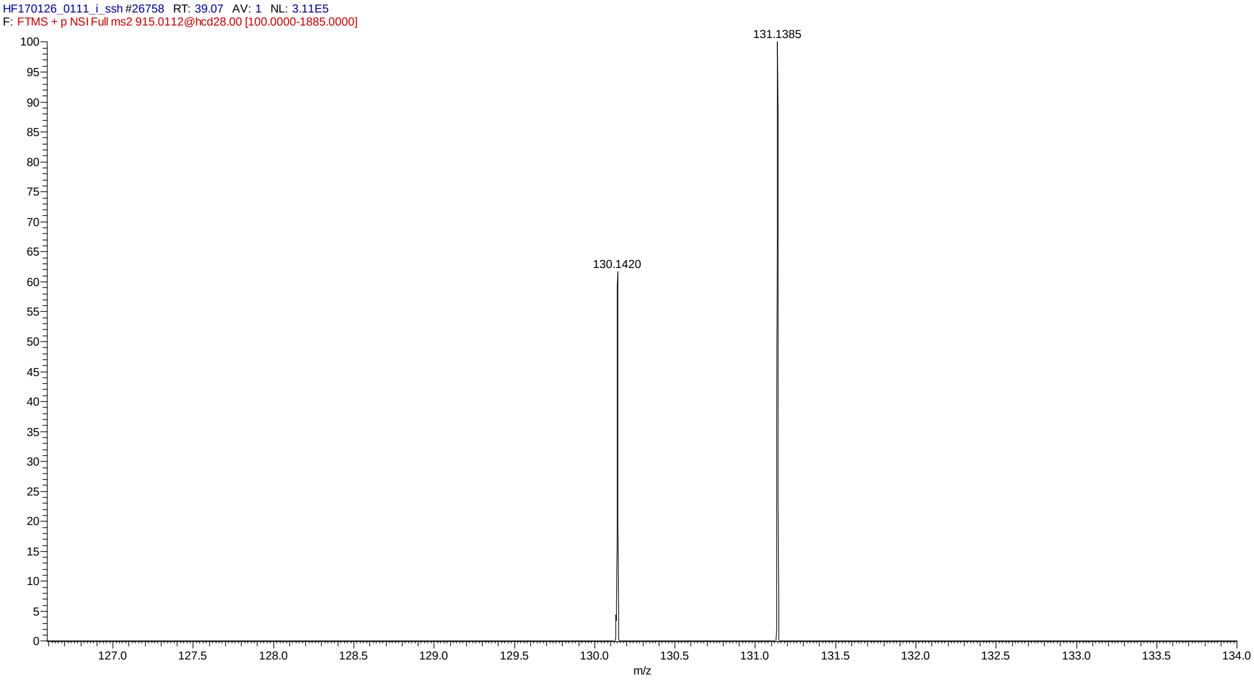

MS/MS Fragmentation of **IVSNASCTTNCLAPLAK**
Found in **G3P_RABIT**, Glyceraldehyde-3-phosphate dehydrogenase OS=Oryctolagus cuniculus GN=GAPDH PE=1 SV=3

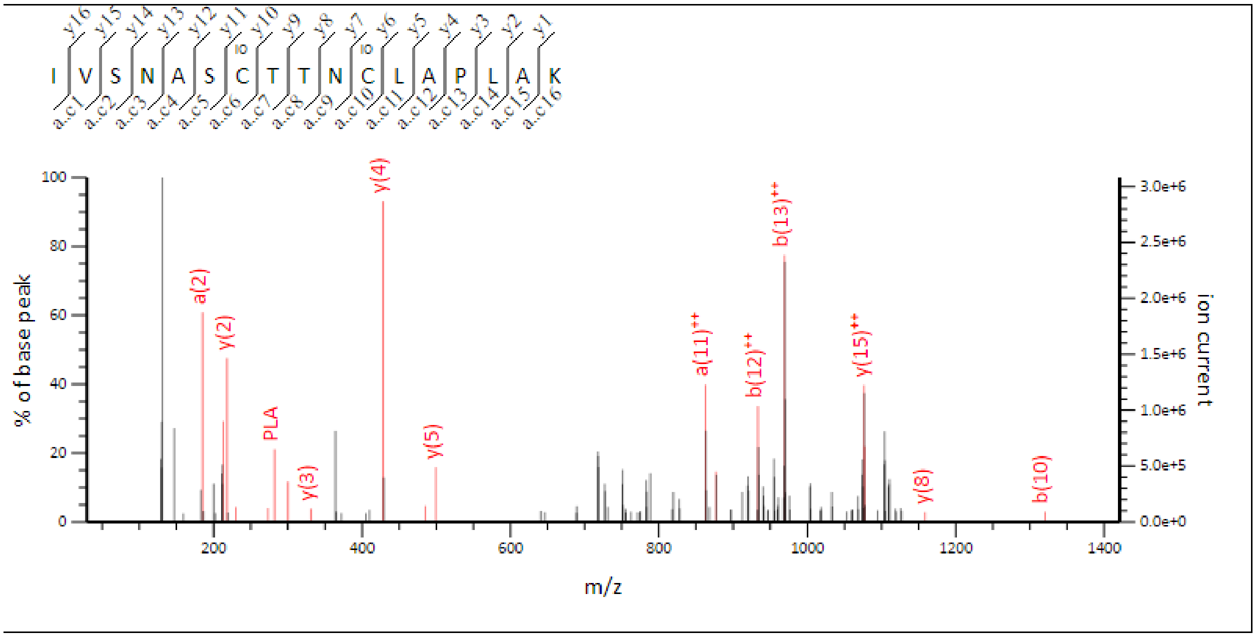

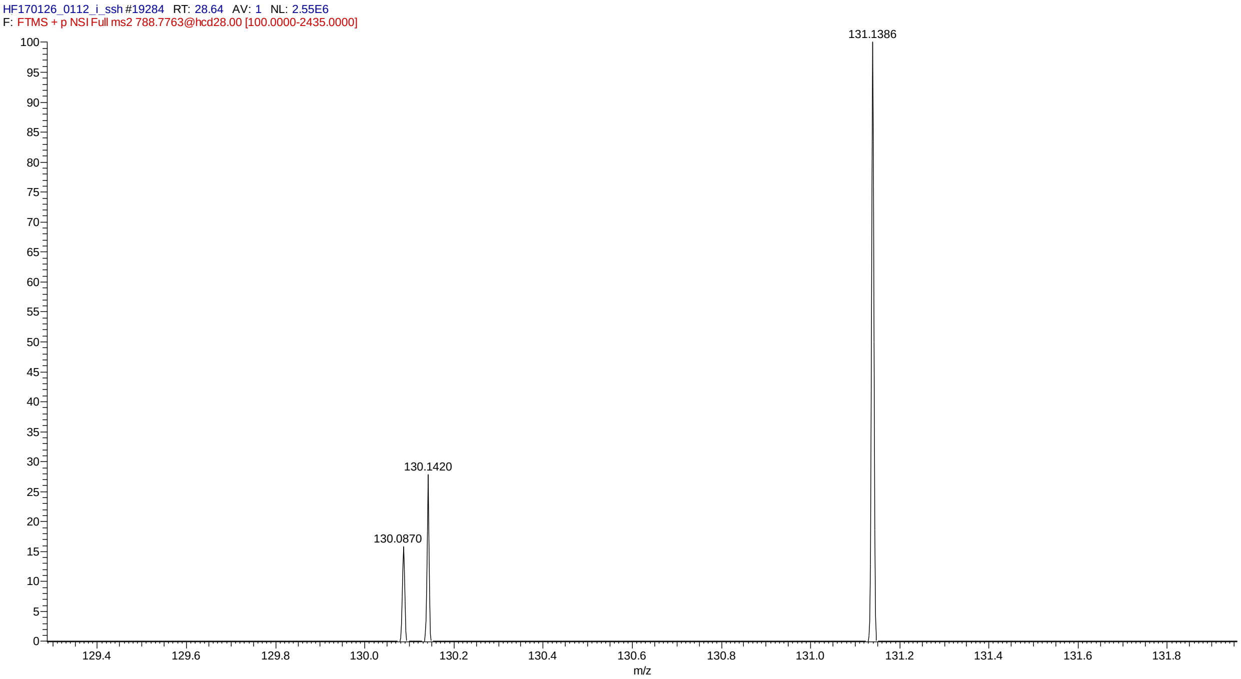

MS/MS Fragmentation of **GILGYTEDQVVSCDFNSATHSSTFDAGAGIALNDHFVK**
Found in **G3P_RABIT**, Glyceraldehyde-3-phosphate dehydrogenase OS=Oryctolagus cuniculus GN=GAPDH PE=1 SV=3

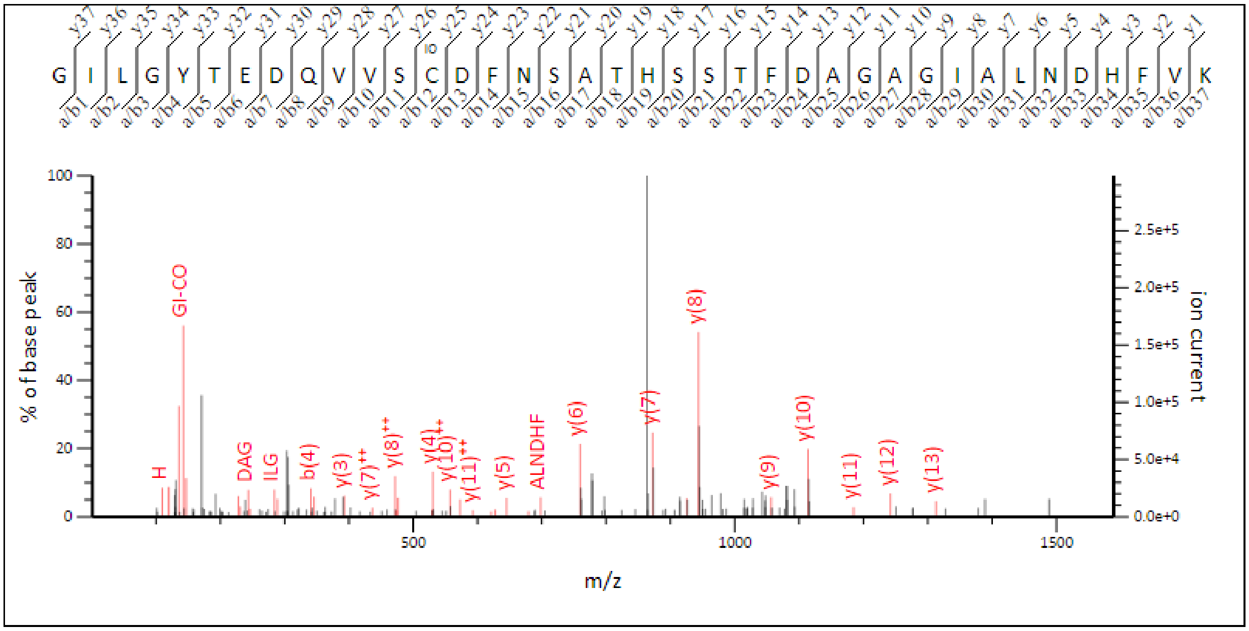
G I L G Y T E D Q V V S C D F N S A T H S S T F D A G A G I A L N D H F V K
% of base peak
ion current
m/z
GI-CO
y(8)
y(7)
y(10)
y(6)
ALNDHF
y(13)
y(12)
y(11)
y(9)
b(4)
ILG
DAG
H

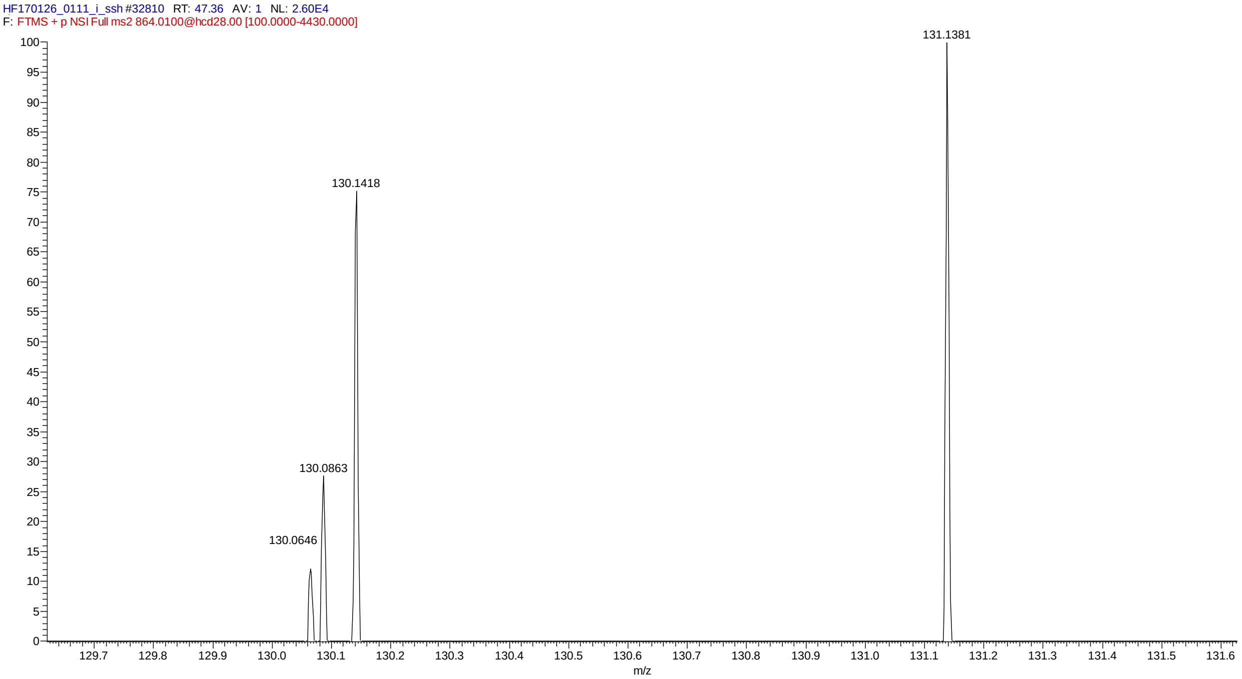
HF170126_0111_i_ssh #32810 RT: 47.36 AV: 1 NL: 2.60E4
F: FTMS + p NSI Full ms2 864.0100@hcd28.00 [100.0000-4430.0000]
131.1381
130.1418
130.0863
130.0646
m/z

Non-reducing SDS-PAGE analysis results

catalase	
red	68.1846327
ox	31.8153673

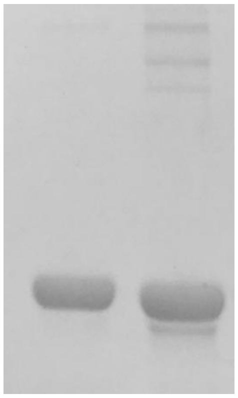

Alkaline Phosphatase	
red	92.3963134
ox	7.60368664

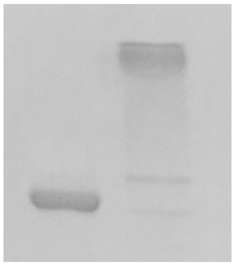

Glyceraldehyde-3-phosphate dehydrogenase	
red	45.621192
rox	54.378808

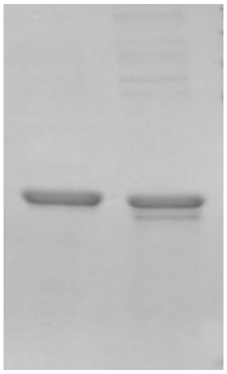

ESM S2

Table S1 Significant changes in protein expression profile after H_2O_2 treatment

Protein name	Protein	Gene name	Expression profile	CV(%)	Significance A
RNA polymerase-binding transcription factor DksA	P0ABS1	dksA	1.93	4.4	8.85E-05
Formate dehydrogenase-O iron-sulfur subunit	P0AAJ5	fdoH	1.78	6.6	2.18E-03
Fumarate reductase iron-sulfur subunit	P0AC47	frdB	2.01	2.4	1.26E-05
Ferric uptake regulation protein	P0A9A9	fur	1.85	4.4	4.79E-04
Galactokinase	P0A6T3	galK	1.63	8.1	3.20E-02
Glutaredoxin-2	P0AC59	grxB	1.84	0.3	6.95E-04
Glutaredoxin-3	P0AC62	grxC	2.10	5.9	1.36E-06
Histidinol-phosphate aminotransferase	P06986	hisC	1.65	2.4	2.44E-02
Hydrogenase-2 operon protein HybA	P0AAJ8	hybA	2.28	0.7	1.27E-08
HTH-type transcriptional regulator IscR	P0AGK8	iscR	1.66	2.8	2.03E-02
Peptide methionine sulfoxide reductase MsrB	P0A746	msrB	1.68	8.7	1.41E-02
NADH-quinone oxidoreductase subunit F	P31979	nuoF	1.82	29.4	9.51E-04
NAD-dependent dihydropyrimidine dehydrogenase subunit PreT	P76440	preT	1.67	2.1	1.79E-02
Phosphoribosylamine--glycine ligase	P15640	purD	1.68	5.4	1.40E-02
D-ribose pyranase	P04982	rbsD	1.61	3.3	4.64E-02
Riboflavin biosynthesis protein RibD;Diaminohydroxyphosphoribosylaminopyrimidine deaminase;5-amino-6-(5-phosphoribosylamino)uracil reductase	P25539	ribD	1.74	2.2	5.24E-03
50S ribosomal protein L31	P0A7M9	rpmE	2.02	11.9	1.01E-05
50S ribosomal protein L36	P0A7Q6	rpmJ	1.85	4.3	5.47E-04
Putative ribosome biogenesis GTPase RsgA	P39286	rsgA	1.62	6.5	3.83E-02
Polyamine aminopropyl transferase	P09158	speE	1.64	10.2	3.11E-02
Periplasmic trehalase	P13482	treA	1.62	2.2	3.98E-02
Thioredoxin-1	P0AA25	trxA	1.64	5.5	3.02E-02
Thioredoxin-2	P0AGG4	trxC	1.84	6.7	6.47E-04
Uncharacterized protein YbeL	P0AAT9	ybeL	1.73	12.7	6.25E-03
Uncharacterized protein YrdD	P45771	yrdD	1.67	20.6	1.85E-02

Table S2 Significantly changed oxidized cysteine fractions in H_2O_2 treated cells. (1)Raw Ox ratio = iodoTMT2/iodoTMT1 (2)Expression profile = iodoTMT4/iodoTMT3 (3)Normalized Ox ratio = oxidized fraction after H_2O_2 treatment (iodoTMT2/iodoTMT4)/oxidized fraction in control (iodoTMT1/iodoTMT3)

Sequence	Proteins	Gene names	Protein names	Control		H_2O_2		Raw Ox ratio (1)	Expression profile (2)	Norm Ox ratio (3)	Norm Ox ratio Significance A p-value
				%Ox	CV(%)	%Ox	CV(%)				
VGDGTQDNLSGCEK	P00963	asnA	Aspartate--ammonia ligase	12.29	5.72	5.29	5.56	0.57	1.32	0.43	3.5E-02
TLEGWGYDYYVFDKVSSPVSTMMACPDGKK	P23827	eco	Ecotin	20.73	9.51	8.76	7.52	0.58	1.37	0.42	2.8E-02
LIDVTTCIGCK	P0AAJ5	fdoH	Formate dehydrogenase-O iron-sulfur subunit	32.55	14.06	14.25	9.93	0.78	1.78	0.44	4.3E-02
MAQLNSQNGVWSCTFVGYCSEVCPK	P0AC47	frdB	Fumarate reductase iron-sulfur subunit	7.97	14.18	3.10	10.04	0.8	2.05	0.39	9.1E-03
SQQVTDACK	P0AC33	fumA	Fumarate hydratase class I, aerobic	34.91	5.31	9.98	11.83	0.43	1.49	0.29	3.4E-05
DKGLILLSCGPYYNVLR	P22256	gabT	4-aminobutyrate aminotransferase GabT	25.81	4.88	9.81	28.07	0.53	1.42	0.38	6.4E-03
ECISENQILK	P0A9C0	glpA	Anaerobic glycerol-3-phosphate dehydrogenase subunit A	30.82	22.67	13.33	2.70	0.58	1.32	0.43	3.7E-02
SLAICTLLDKPSR	P0A9M2	hpt	Hypoxanthine phosphoribosyltransferase	43.85	22.73	15.33	28.53	0.49	1.25	0.35	1.7E-03
TGIVHYDKDVCTGCR	P0AAJ8	hybA	Hydrogenase-2 operon protein HybA	26.32	2.36	6.61	3.56	0.57	2.28	0.25	1.7E-06
VLVCVPVGATQVERR	P0A9X4	mreB	Rod shape-determining protein MreB	20.12	20.25	8.02	13.25	0.59	1.41	0.4	1.3E-02
KHSICPSGKR	P0A9L5	ppiC	Peptidyl-prolyl cis-trans isomerase C	29.28	3.75	12.91	5.47	0.63	1.43	0.44	4.6E-02
CHPFFTGK	P0A7M9	rpmE	50S ribosomal protein L31	10.08	15.76	4.02	14.32	0.91	2.29	0.4	1.3E-02
GEDVVVPGLFAVGEIACVSVHGANR	P0AC41	sdhA	Succinate dehydrogenase flavoprotein subunit	16.85	13.28	7.18	20.62	0.56	1.32	0.43	3.1E-02
DDLPVVIDFWAPWCGPCR	P0AGG4	trxC	Thioredoxin-2	15.33	7.85	6.37	16.49	0.76	1.84	0.42	2.2E-02
HGANLILLDISPEIEKLADELCGR	P37440	ucpA	Oxidoreductase UcpA	33.11	19.35	13.89	20.89	0.66	1.51	0.42	2.5E-02
CHFHLPIYTPEVLTLCPK	P0AAT9	ybeL	Uncharacterized protein YbeL	9.91	8.20	4.40	15.02	0.84	1.88	0.44	5.0E-02
AGIEAANFPFCTIEPNTGVVPMPDPR	P0ABU2	ychF	Ribosome-binding ATPase YchF	6.26	26.31	2.68	21.48	0.53	1.21	0.43	3.2E-02
LVIECTGMADPGPIIQTFFSHEVLCQR	P24203	yjiA	Uncharacterized GTP-binding protein YjiA	12.72	15.38	5.54	9.81	0.62	1.37	0.44	4.1E-02
